# Supplementary material for: The occurrence of ansamers in the synthesis of cyclic peptides
Source: Nat Commun. 2022 Oct 30;13:6488. doi: 10.1038/s41467-022-34125-8 (PMC9618573; doi:10.1038/s41467-022-34125-8)
Supplement: Supplementary file 1 — Supplementary Information [file 41467_2022_34125_MOESM1_ESM.pdf]

Supplementary Information

The occurrence of ansamers in the synthesis of cyclic peptides

Guiyang Yao[1,2, ‡], Simone Kosol[1, ‡], Marius T. Wenz[3, ‡], Elisabeth Irran,[1] Bettina G. Keller[3], Oliver Trapp[4] and Roderich D. Süssmuth[1*]

1 Institut für Chemie, Technische Universität Berlin, Strasse des 17. Juni 124, 10623 Berlin, Germany.

2 Center for Innovative Drug Discovery, Greater Bay Area Institute of Precision Medicine (Guangzhou), School of Life Sciences, Fudan University, PR China.

3 Department of Biology, Chemistry, Pharmacy, Freie Universität Berlin, Arnimallee 22, 14195 Berlin, Germany.

4 Department of Chemistry and Pharmacy, Ludwig-Maximilians-University, Butenandtstr. 5-13, 81377 Munich, Germany, Max-Planck-Institute for Astronomy, Königstuhl 17, 69117 Heidelberg, Germany.

[‡] These authors contributed equally to this work.

*e-mail: suessmuth@chem.tu-berlin.de

Contents

1. Supplementary Figures 4

2. Supplementary Tables 21

3. Supplementary Methods 25

3.1 Synthesis protocols and characterization data 25

3.1.1 Reagents, Solvents and Chromatographic Conditions 25

3.1. 2 Abbreviations 25

3.1.3 Variable temperature NMR (VT-NMR) 26

3.1.4 NMR assignment and structure calculation of desulfurized macrolactam 5 26

3.1.5 Structure desulfurized macrolactam 5 27

3.1.6 Analytical Methods 27

3.1.7 Experimental 27

3.1.7.1 General protocol 27

3.1.7.2 I_2-mediated thioether formation 28

3.1.7.3 Cleavage from solid support 28

3.1.7.4 Monitoring of Peptide Coupling and Capping 28

3.1.7.5 Macrolactamization 29

3.1.7.6 Deprotection 29

3.1.8 Synthesis and characterization data 29

3.1.8.1 Synthesis of monocyclic peptides 2a-2d and 3a-3d 29

3.1.8.2 Synthesis of bicyclic peptides 4a and 4b 33

3.1.8.3 Amino acid analysis of 4a and 4b (Marfey's reagent) 34

3.1.8.4 Heating experiment of 4a and 4b 35

3.1.8.5 Desulfurization of 4a and 4b 35

3.1.9 CD spectroscopy 35

3.2 Molecular dynamics simulations 37

3.2.1 Setup of the MD simulations 37

3.2.1.1 Parametrization of the peptides 4a, 4b, 3b and 3c 37

3.2.1.2 Parameters for MD simulation of the peptides 4a, 4b, 3b and 3c 37

3.2.2 Methods: Analyses 38
3.2.2.1 Plane angle analysis 38
3.2.2.2 Distance calculations 40
3.2.2.3 Hydrogen bonds 40
3.2.2.4 Linear correlations between time series of observables 42
3.2.2.5 RMSD / RMSF calculations 42
3.3 NMR spectra 46
3.4 X-Ray 53
4 Supplementary Reference 68

1. Supplementary Figures

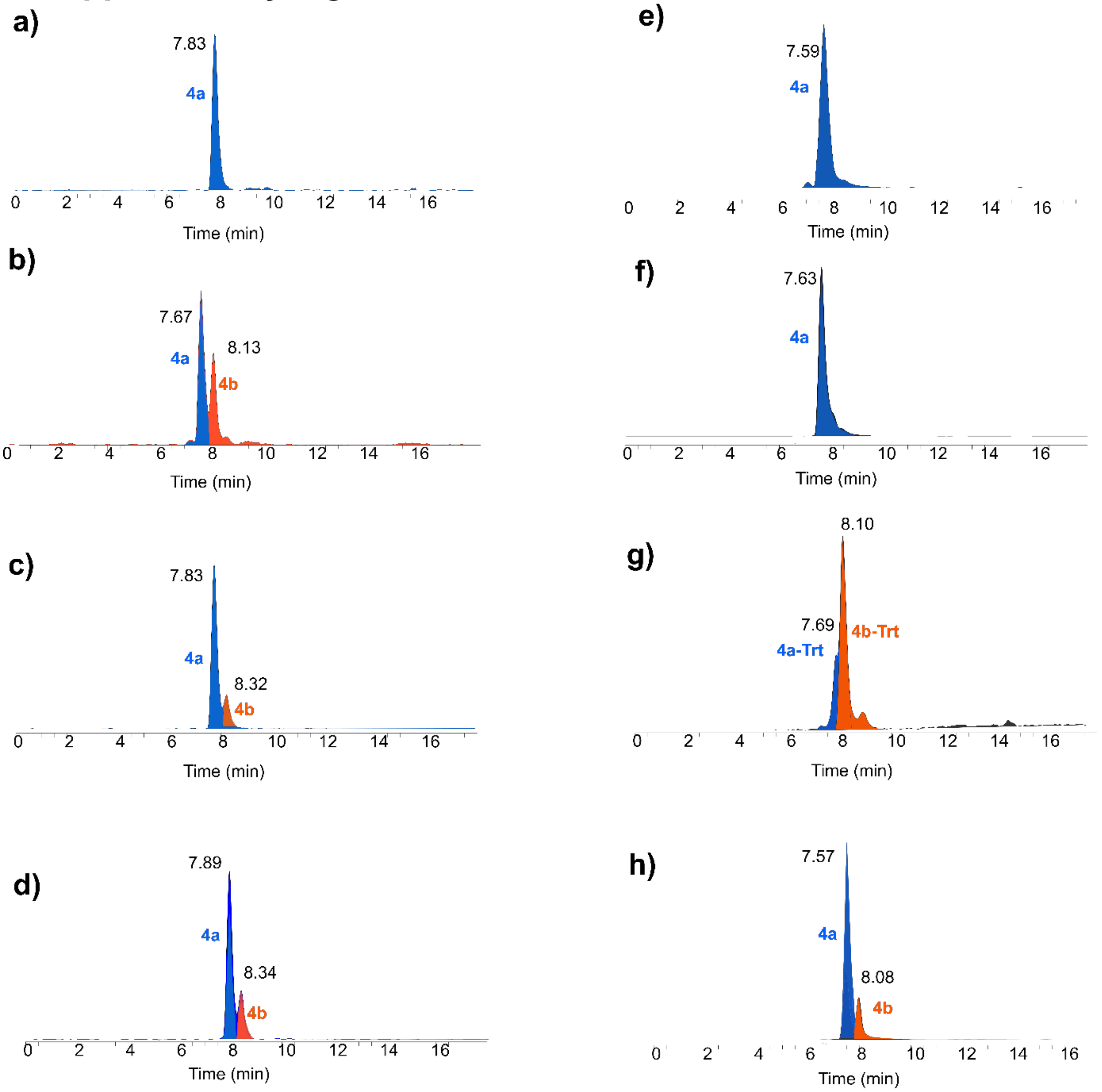

Supplementary Figure 1: LC-MS of precursor peptides. LC-MS results of the macrolactamization of monocyclic precursors **2a-2d** and **3a-3d**. The gradient of a)-f) and h) is gradient D, the gradient of g) is gradient B.

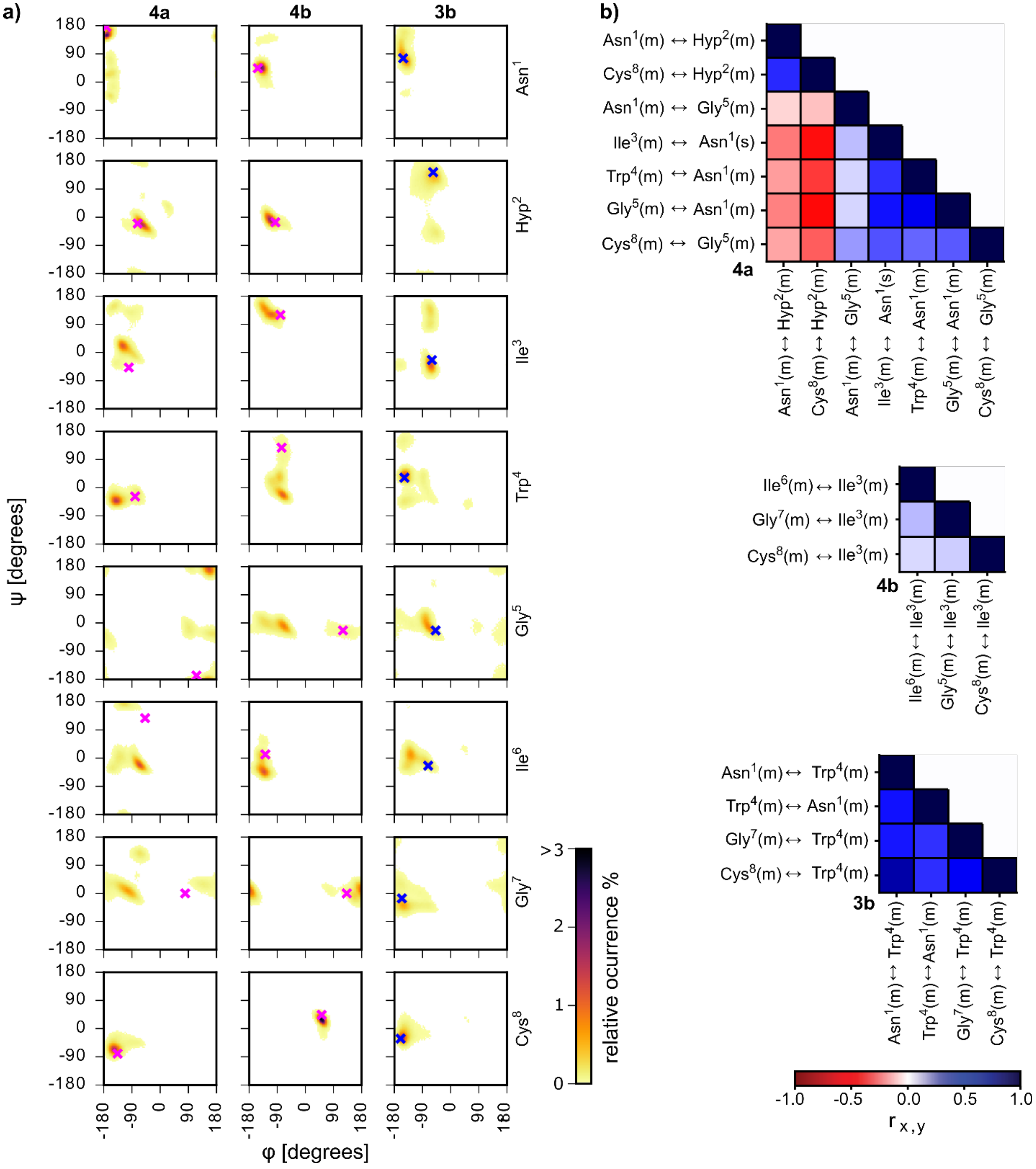

Supplementary Figure 2: Dihedral angle and hydrogen bond analyses of MD structural ensembles. a) Ramachandran plots for the molecules **4a**, **4b** and **3b**. Reference values taken from the crystal structures are indicated as pink crosses. Reference values taken from the highest-probability structure of the MD ensemble of **3b** are indicated as blue crosses (see 3.2.2.9). The backbone angle distributions are normalized referring to the simulation length (20 µs). b) Pearson correlation coefficients ($r_{x,y}$) for the hydrogen bonds with a population greater than 10% on average over all replica (n=20). Values close to |1| indicate a strong correlation (positive, blue) or anti-correlation (negative, red). 'm' denotes the main chain, 's' the side chain of the respective residue.

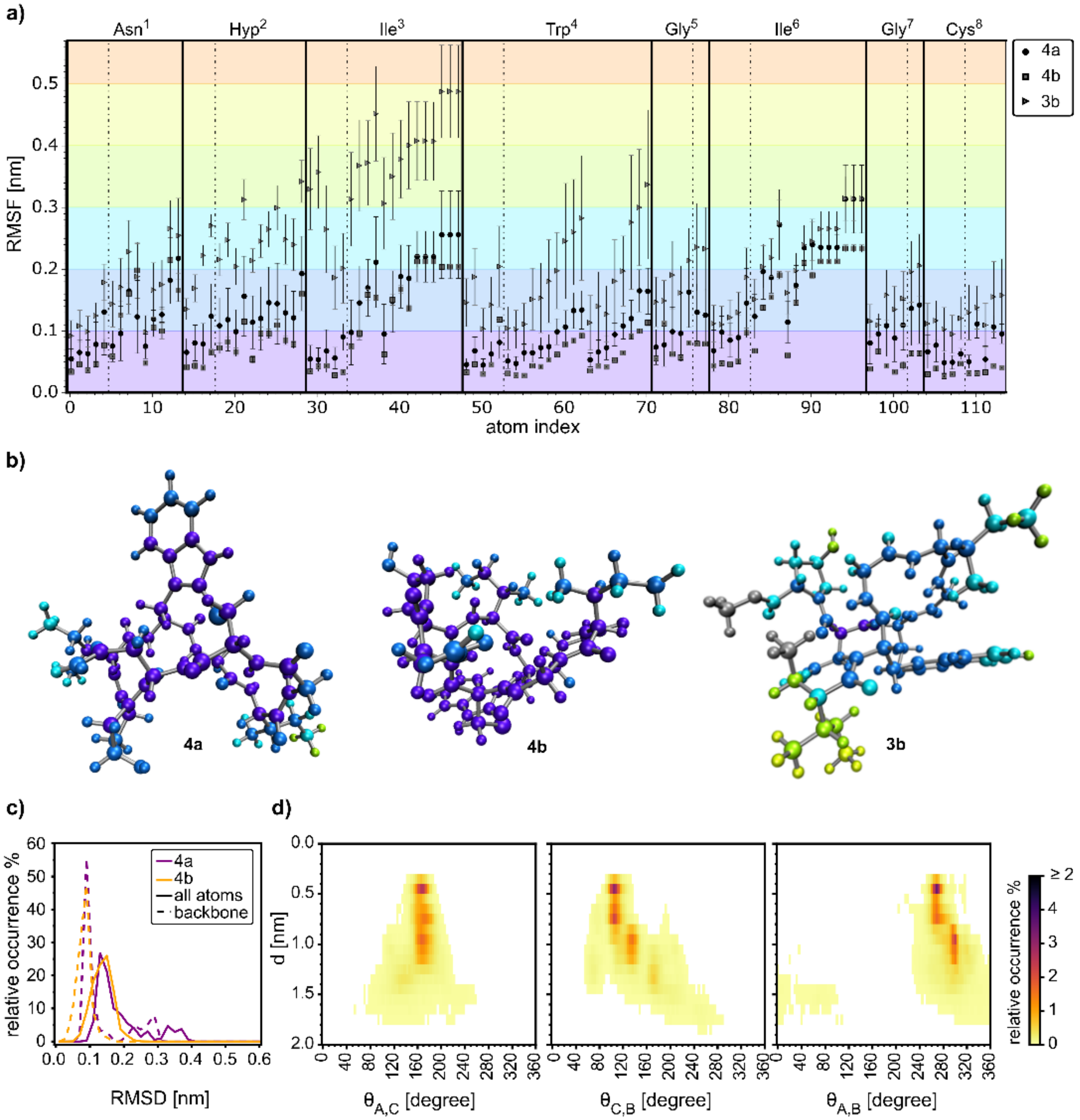

Supplementary Figure 3: Comparison of MD data with crystal structures. a) All-atom RMSF for **4a, 4b** and **3b** with reference to the respective crystal structure (**4a**, **4b**) or one structure of the subset containing the highest probability structure of the MD simulations (**3b**). In each system, the all-atom RMSF was calculated individually for 20 replicas (1 µs simulation time each). The resulting arithmetic mean (points) and standard deviation (error bars) over all replicas (n=20 representing 20 µs simulation time) are shown for each atom grouped according to the amino acid residues. The groups are separated by solid lines. The dotted lines separate 'main-chain' atoms (left) from 'side-chain' atoms (right), where 'main chain' is defined as 'N', 'H' (amide), 'CA', 'C' and 'O' for each residue following the GROMACS convention. For all molecules, the same atom order was applied: N, CA, C, O, for main chain atoms, followed by the carbon atoms along the side chain and hetero-atoms and lastly, the hydrogens. For the atom indices,

please refer to the methods section and supplied structure files ('RMSF calculations'). Caps were neglected for RMSF calculations. b) Structures of **4a**, **4b** and **3b**. The atoms are colored according to the respective RMSF values: purple: 0.0 - 0.1 nm, blue: 0.1 - 0.2 nm, cyan: 0.2 - 0.3 nm, green: 0.3 - 0.4 nm, yellow: 0.4 - 0.5 nm, orange: 0.5 - 0.6 nm. c) Distribution of the all-atom RMSD (solid line) and the backbone RMSD (dotted line) averaged over all replicas (n=20, 20 µs in total) for **4a** (purple) and **4b** (orange), respectively. d) Joint probability distribution normalized to the simulation length of 20 µs, for the distance between the C- and the N-terminus in **3b** and the angles between the planes $\theta_{A,B}$, $\theta_{A,C}$, $\theta_{C,B}$ defined in our model to assess the spatial orientation of ring A (Cys[8]-Asn[1]-Hyp[2]-Ile[3]-Trp[4]), ring B (Trp[4]-Gly[5]-Ile[6]-Gly[7]-Cys[8]) and the tryptathionine bridge. For the definition of the model, please refer to the methods section ('Plane angles'). The C-N-terminus distance was measured between the carbon atom of the carbonyl group in the C-terminus (-**C**O-OMe) and the nitrogen atom in the N-terminus (-**N**H-Me).

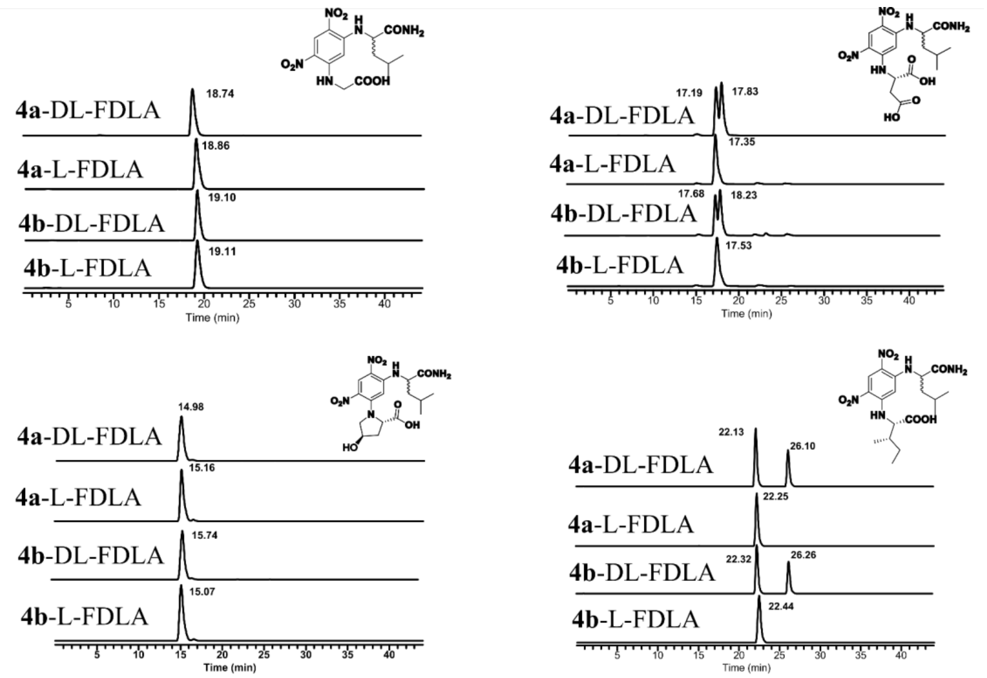

Supplementary Figure 4: Marfey analytics of amino acids in peptides 4a and 4b. HPLC-MS chromatograms of the amino acid analysis of peptides **4a** and **4b** (total hydrolysis followed by modification with Marfey's reagent, treated either with L-FDLA or the racemic DL-FDLA reagent (HPLC gradient E).

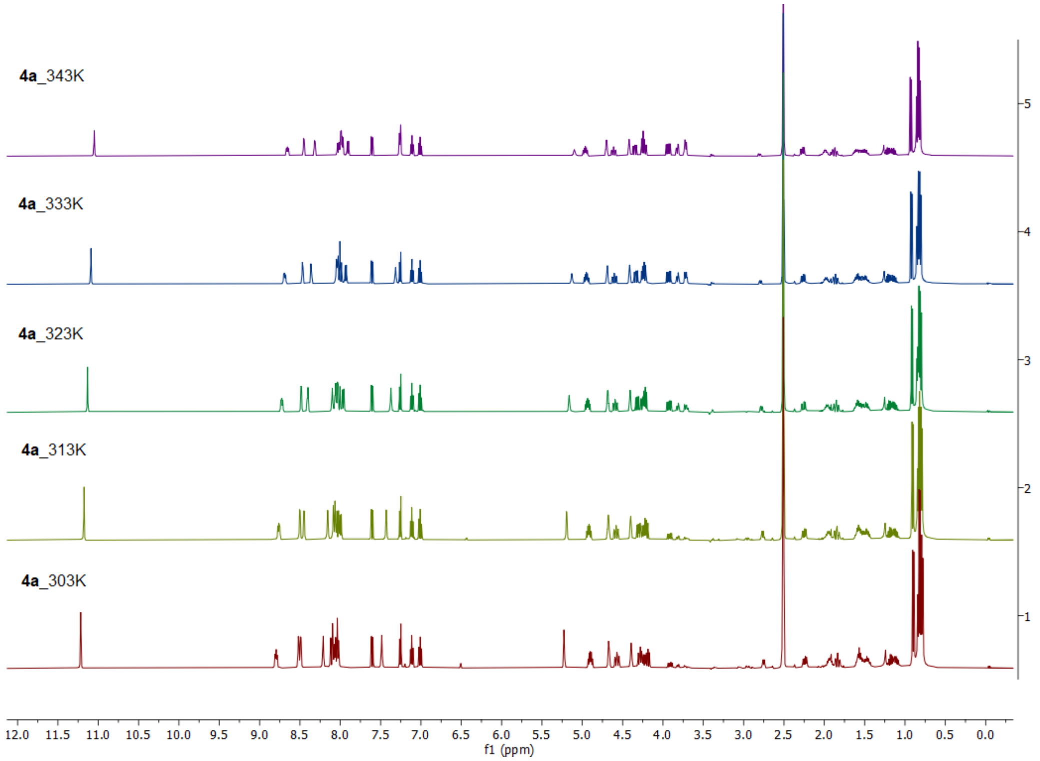

Supplementary Figure 5: VT-NMR of isomer 4a. Variable-temperature NMR spectroscopy study of peptide **4a** at temperatures ranging from 303K to 343K.

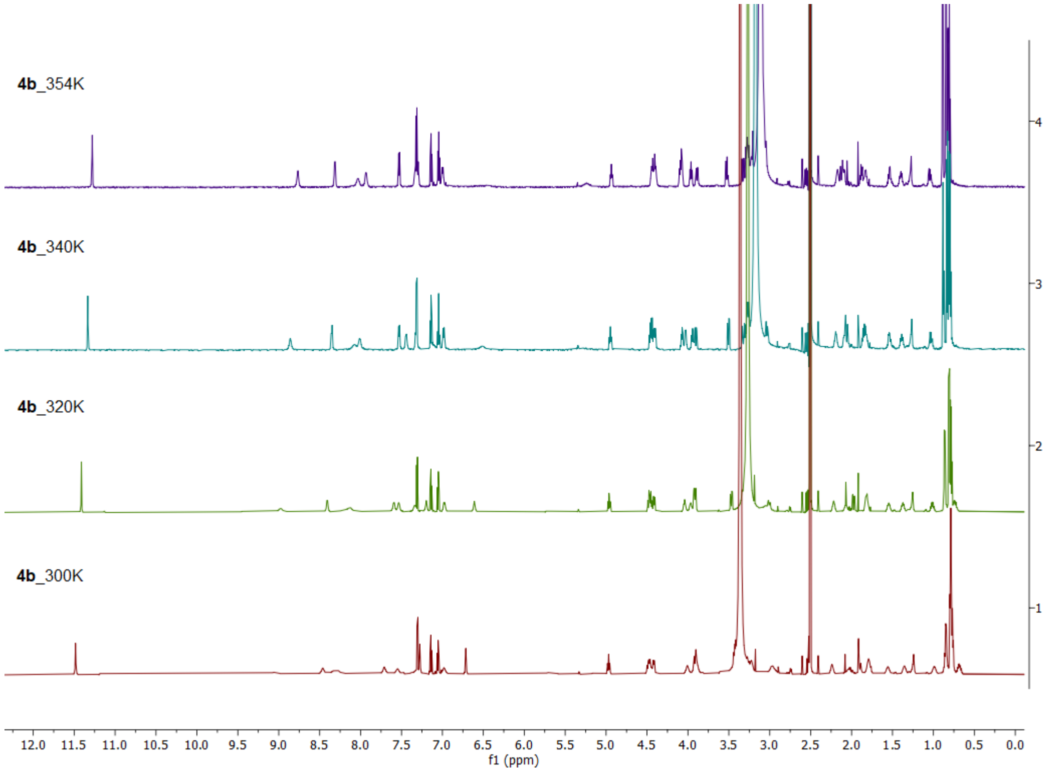

Supplementary Figure 6: VT-NMR of isomer 4b. Variable-temperature NMR spectroscopy study of peptide **4b** at temperatures ranging from 300K to 354K.

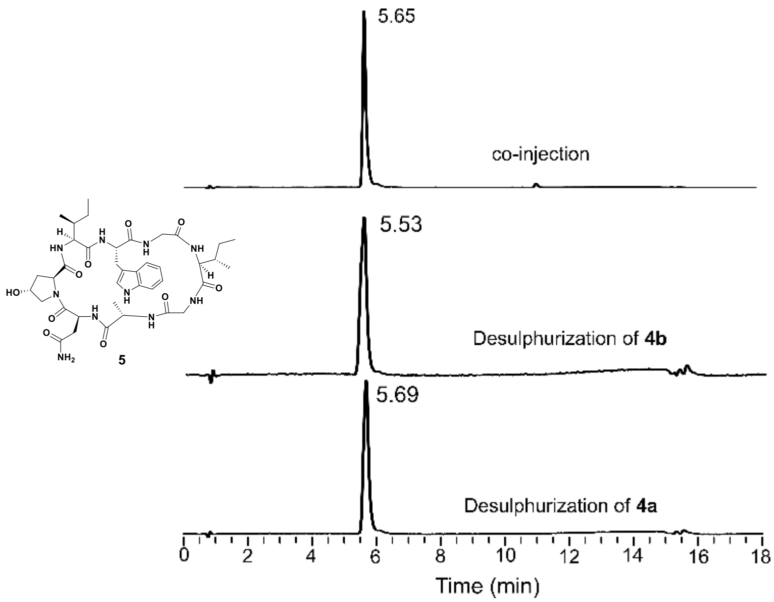

Supplementary Figure 7: Desulfurization of the isomers. LCMS chromatograms of the desulfurization reaction of bicyclic **4a** and **4b** (Raney-Ni in MeOH, 5 h) yielding compound **5**.

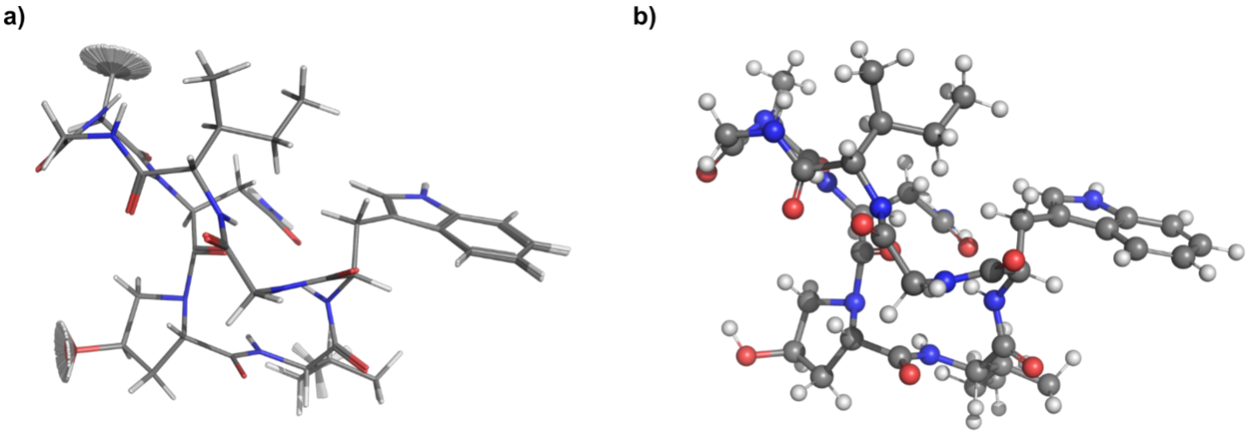

Supplementary Figure 8: Solution structure of desulfurized isomers. NMR-based solution structure of the desulfurized macrolactam **5**. A) The 100 lowest energy structures of desulfurized **4a** and **4b** have an RMSD of 0.01. B) Stick and ball model of the average state. The atoms are colored according to their type: grey: carbon, white: hydrogen, red: oxygen, blue: nitrogen.

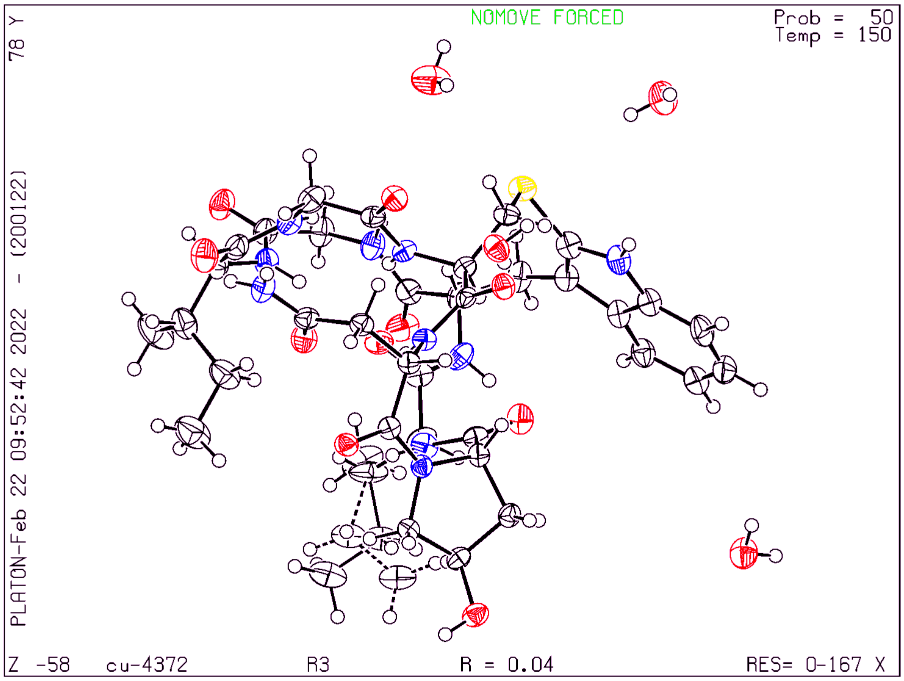

Supplementary Figure 9: Crystal structure of 4b. Crystal structure of **4b** visualized by PLATON. The electron density map shows two conformations for the ethyl group of Ile[3] (the second conformation is indicated with dashed bonds). Nitrogen atoms are colored blue, oxygen atoms are shown in red, sulfur atoms are yellow, carbon atoms are depicted as white and black ellipsoids and hydrogens are shown as small white circles.

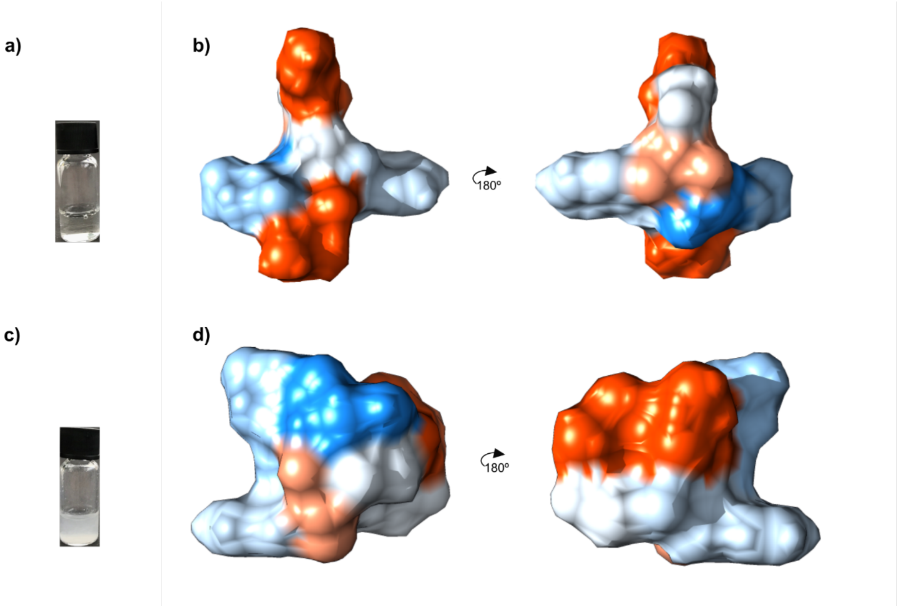

Supplementary Figure 10: Solubility and overall shape of isomers. Compounds a) **4a** and c) **4b** dissolved in water (at 2 mM concentration). Surface representations of b) **4a** and d) **4b**, respectively. Coloring according to the amino acid hydrophobicity following Kyte et al. [1] (hydrophobic residues are colored in orange and polar residues in blue).

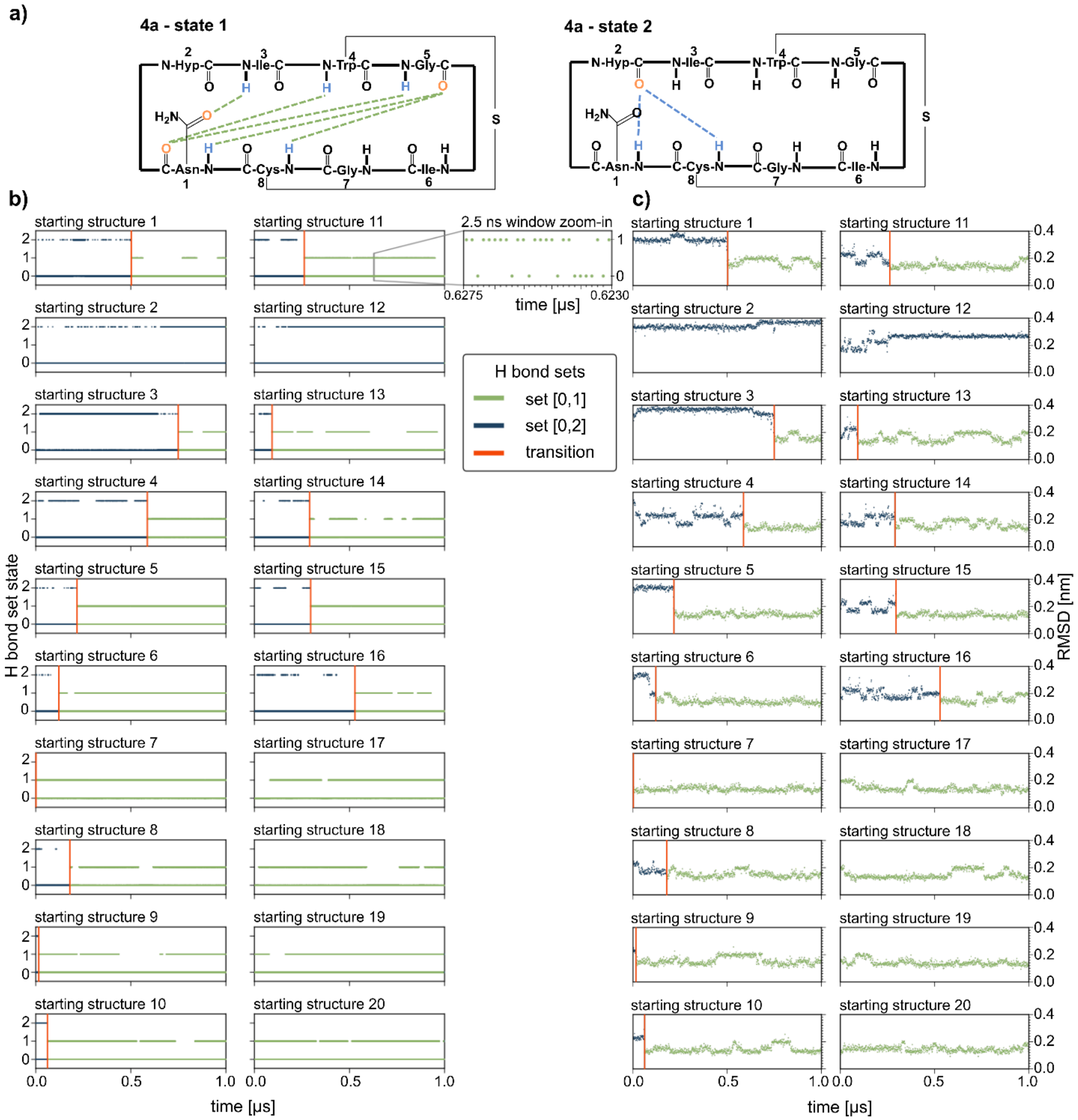

Supplementary Figure 11: Hydrogen bond sets observed in MD simulations. (a) Hydrogen bond sets '1' (olive) and '2' (blue) for **4a**. Hydrogen bond set '1' denotes the hydrogen bonds that agree with the crystal structure of **4a**: Asn^1(m)-Gly^5(m), Ile^3(m)-Asn^1(s), Trp^4(m)-Asn^1(m), Gly^5(m)-Asn^1(m), Cys^8(m)-Gly^5(m). Hydrogen bond set '2' denotes the hydrogen bonds that were identified as mutual exclusive to hydrogen set '1' (see Pearson correlations, Supplementary Figure 2b): Asn^1(m)-Hyp^2(m), Cys^8(m)-Hyp^2(m). (b,c) Time series for the trajectories of **4a** either expressed in b) hydrogen bond set existences or c) RMSD values. The coloring in (b) and (c) is based on the existence of the hydrogen bond sets: set '1' or set '0': olive, set '2' or set '0' : blue. Hydrogen bond set '0' denotes all short-term transitions out of the two conformations. The inset shows that all states are exclusive to each other. Vertical red lines denote the transition between the hydrogen bond sets '2' and '1' representing the transition

between the starting structure of the MD simulations and the crystal structure. For further details, please refer to section 3.2.2.10.

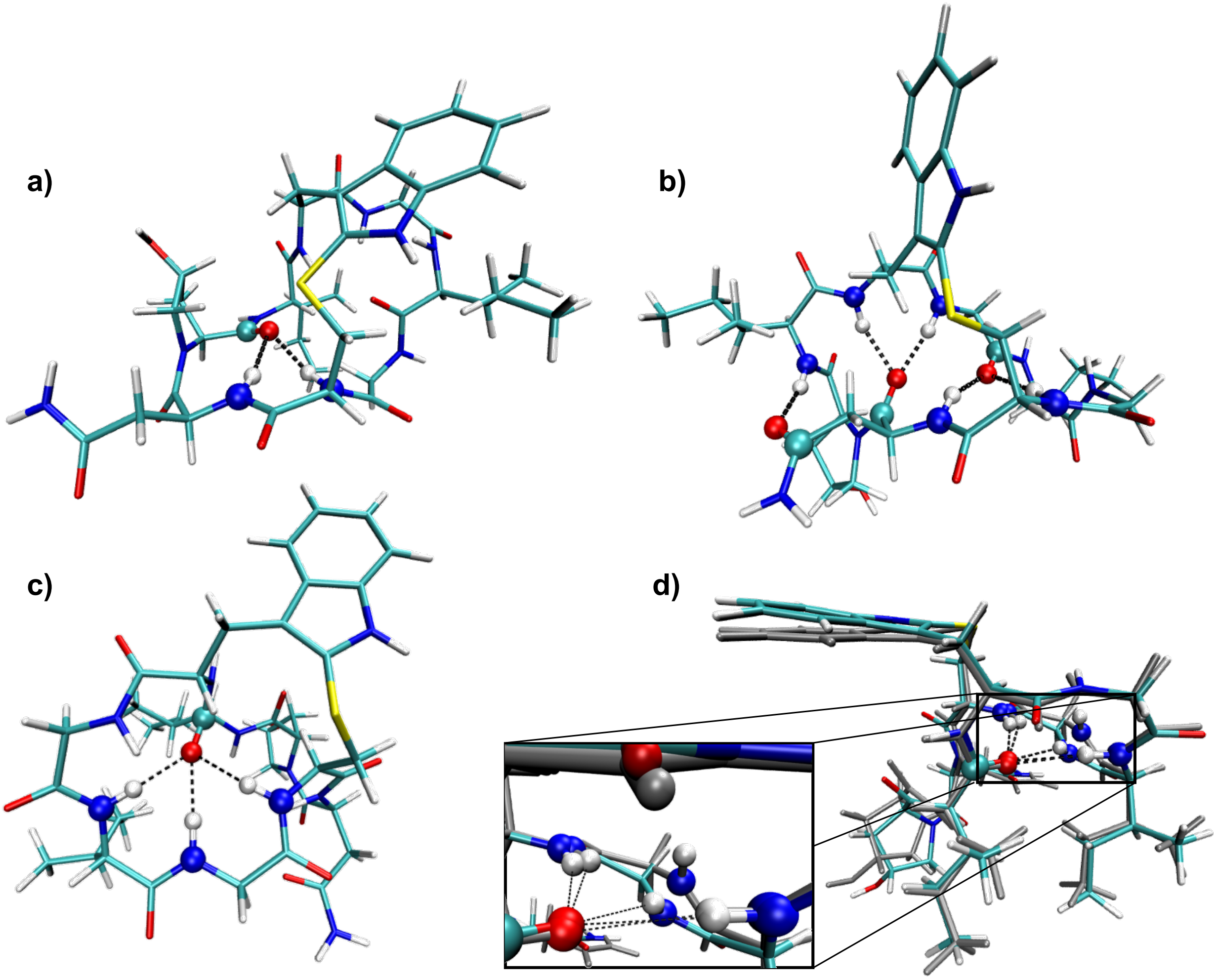

Supplementary Figure 12: MD starting structures. Starting structures of the production MD runs for **4a** with a) worst and b) best agreement to the crystal structure. As measure for the agreement between starting structures and the crystal structure, the RMSD was considered. The RMSD was calculated on all atoms after a least-square fit to the backbone. Important hydrogen bonds are highlighted and correspond to the mutual exclusive hydrogen bond sets 1 and 2. Set 2 (a) denotes the hydrogen bonds: Asn^{1}(m)-Hyp^{2}(m), Cys^{8}(m)-Hyp^{2}(m). Set 1 (b) denotes the hydrogen bonds that agree with the crystal structure of **4a**: Asn^{1}(m)-Gly^{5}(m), Ile^{3}(m)-Asn^{1}(s), Trp^{4}(m)-Asn^{1}(m), Gly^{5}(m)-Asn1(m), Cys^{8}(m)-Gly^{5}(m). 'm' denotes the main chain of the respective residue, 's' denotes the side chain. For the assignment of the atoms, please refer to the methods section ('Hydrogen bonds').
(c) Structure for **4b** taken from the MD ensemble that exhibits the hydrogen bonds Ile^{6}(m)-Ile^{3}(m), Gly^{7}(m)-Ile^{3}(m) and Cys^{8}(m)-Ile^{3}(m). (d) Overlay of the structure shown in (c) with the crystal structure of **4b** (grey). The region of the hydrogen bonds is shown in the details view. Atoms are colored according to their type: red: oxygen, blue: nitrogen, cyan: carbon, white: hydrogen, yellow: sulfur.

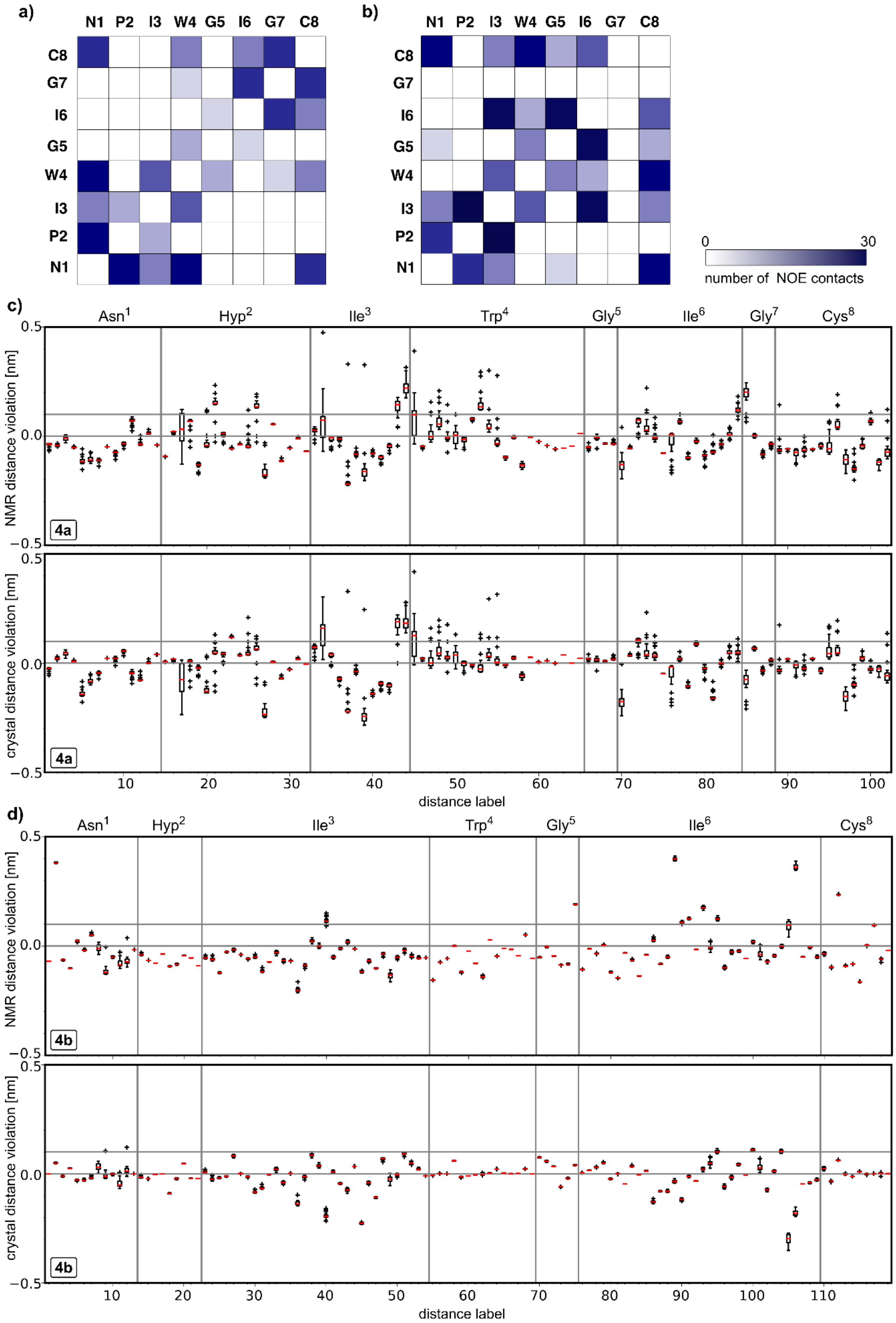
a)
N1 P2 I3 W4 G5 I6 G7 C8
b)
0
30
number of NOE contacts
c)
Asn1 Hyp2 Ile3 Trp4 Gly5 Ile6 Gly7 Cys8
NMR distance violation [nm]
crystal distance violation [nm]
4a
distance label
d)
Asn1 Hyp2 Ile3 Trp4 Gly5 Ile6 Cys8
4b

Supplementary Figure 13: Comparison of isomer NOE data and agreement with MD simulations. Distinct NOE patterns in contact maps of a) **4a** and b) **4b**. Distance violations of proton distances in the MD simulations compared to the NMR NOE data or crystal structures of **4a** c) and **4b** d). In each case, the same set of proton-proton distances was derived from NMR experiments and the crystal structures. The distances from MD were calculated as averages (see eq. 7) per replica representing 1 µs of simulation time respectively (see 3.2.2.11). The distribution of the resulting violations over all replicas (n=20) is shown as follows: the box represents 50% of the data points from the first to the third quartile with the median highlighted in red. The whiskers extend from the box by 1.5-fold of the inter-quartile range. Outliers are marked as black '+'.

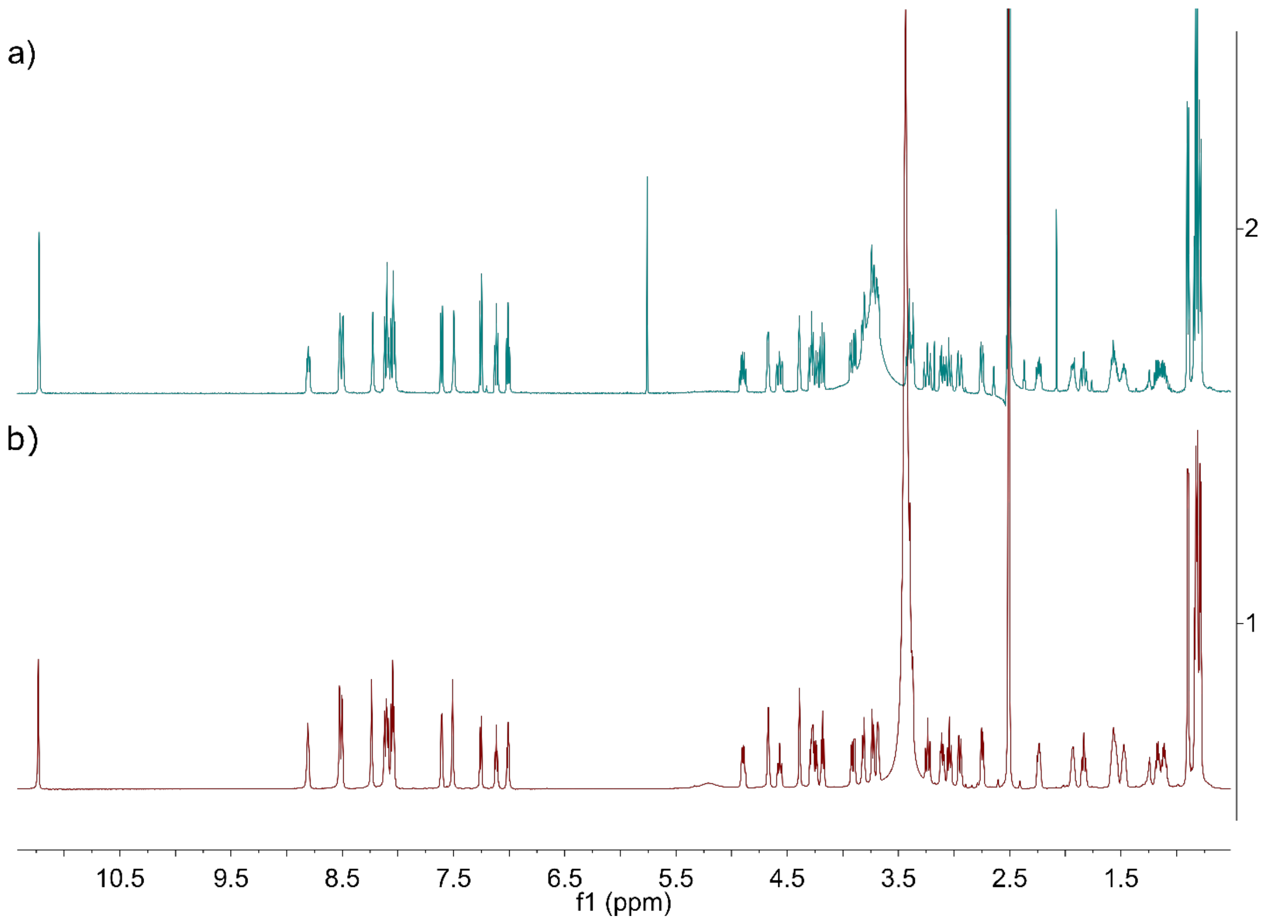

Supplementary Figure 14: Comparison of synthesis product 4a from different precursors. Overlay of ^{1}H NMR spectra of synthetic **4a,** a) **4a** was produced after macrolactamization of **3c** and b) **4a** was produced after macrolactamization of **2b**.

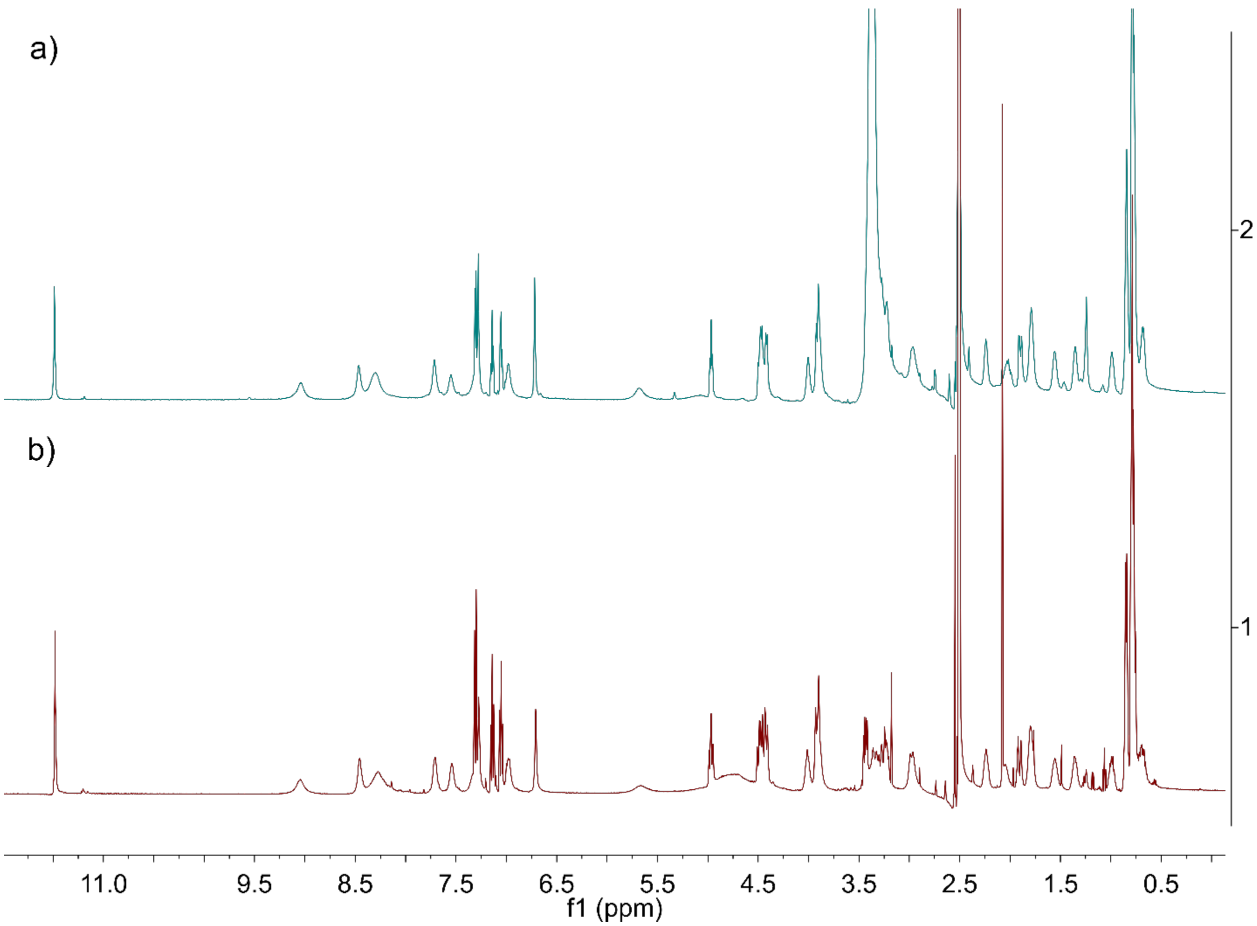

Supplementary Figure 15: Comparison of synthesis product 4b from different precursors. Overlay of ^{1}H NMR spectra of synthetic **4b**, a) **4b** was produced after macrolactamization of **3c** and **4b** was produced after macrolactamization of **2b**).

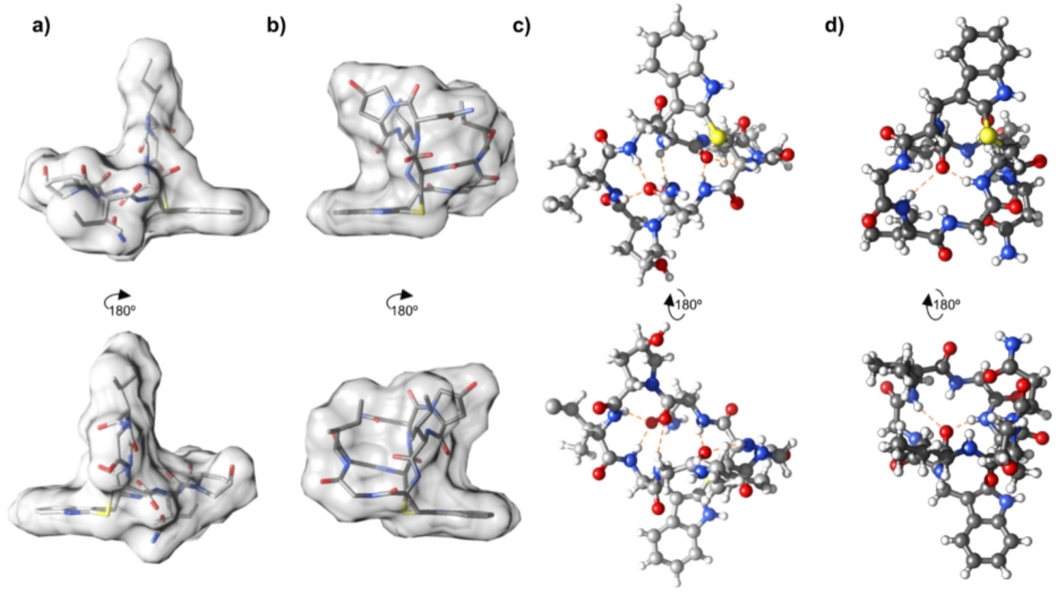

Supplementary Figure 16: Comparison of crystal structures 4a and 4b. Surface representation of the crystal structures of a) **4a** and b) **4b**. **4a** has a volume of 744.1 Å^3 and 598.0 Å^2.**4b** has a volume of 757.6 Å^3 and a surface area of 557.9 Å^2. Ball and stick models of crystal structures of c) **4a** and d) **4b** with hydrogen bonds shown as dashed orange lines. Atoms are colored according to their type: red: oxygen, blue: nitrogen, grey: carbon, white: hydrogen, yellow: sulfur.

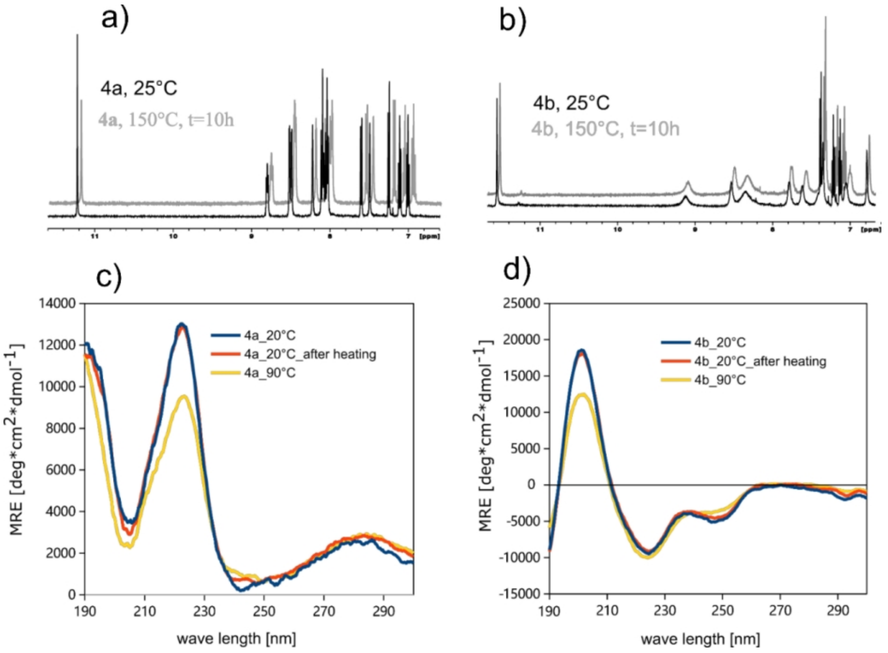

Supplementary Figure 17: Spectroscopic evaluation of heating experiments. a) ^{1}H NMR evidence for the lack of interconversion in **4a** upon heating and b) **4b;** c and d) CD spectroscopy study of peptides **4a** and **4b** at different temperatures.

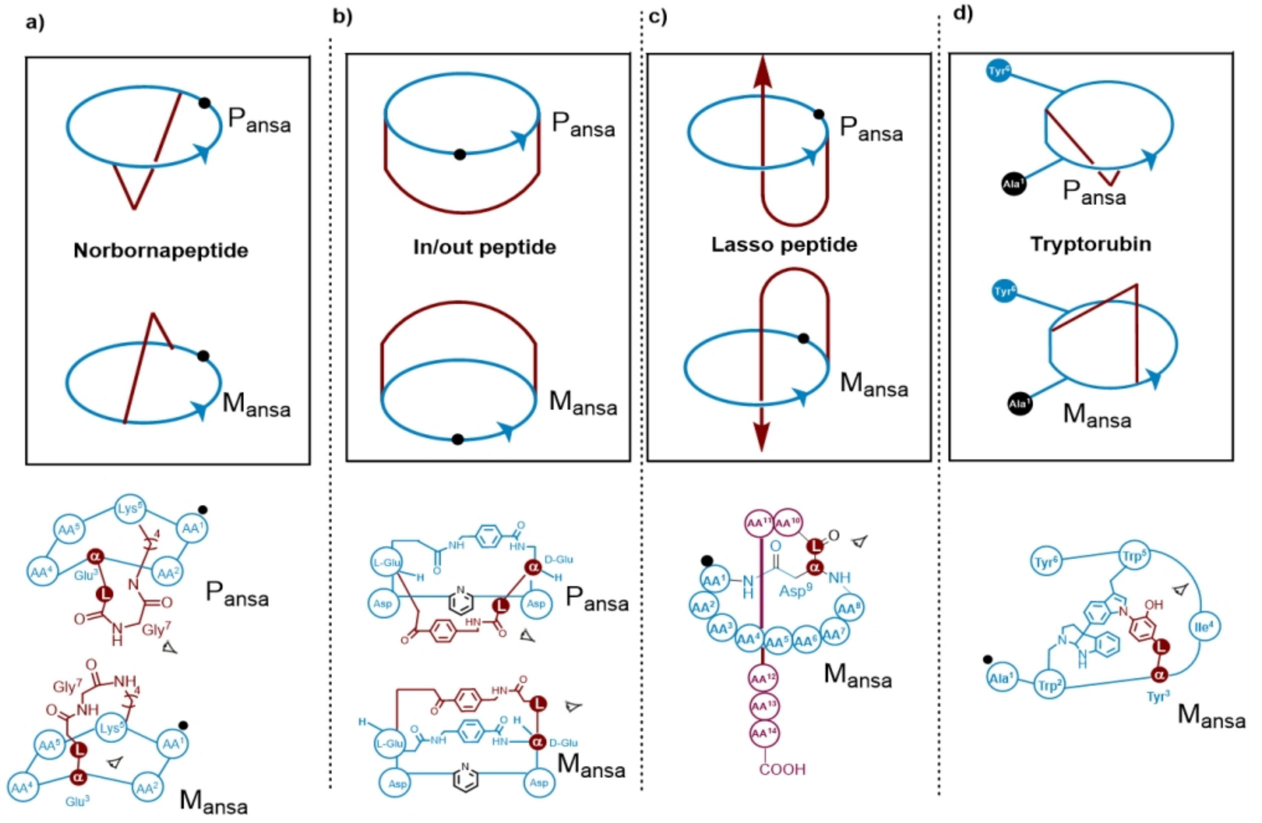

Supplementary Figure 18: Examples of cyclic peptides and proposed application of the ansamer concept and the *P*$_{ansamer}$/*M*$_{ansamer}$ nomenclature. Main ring of the peptide (blue) with starting moiety/amino acid (black dot) forms the imaginary plane with proposed preferred directionality (blue arrow head). Bridge (red) above or below the main ring. Examples include a) norbornapeptide-type [2], b) in/out peptides [3], c) lasso peptides [4] and d) tryptorubin [5]. a) **Norbornapeptides**: For the drawn example there is an analogous situation as for the amanitin peptides. Main ring: head-to-tail cyclic peptide. Preferred directionality according to the directionality of the N- and C-terminus. b) **in/out peptides**: Determination of the main ring and assignment of the bridges leads to assignment of the P$_{ansa}$/M$_{ansa}$ descriptor. c) **Lasso peptides**: Main ring: cyclopeptide (example herein Gly^1-β-Asp^9). Preferred directionality according to the N- and C-terminus. Bridge: peptide tail (example herein: Ala^{10} to Asn^{19}). d) **Tryptorubin**: Main ring replaced here by linear peptide from N- to C-terminus (compare a related view on the vancomycin structure [6]) In this example the bridge connects theTyr^3 with Trp^5 sidechains of the main peptide.

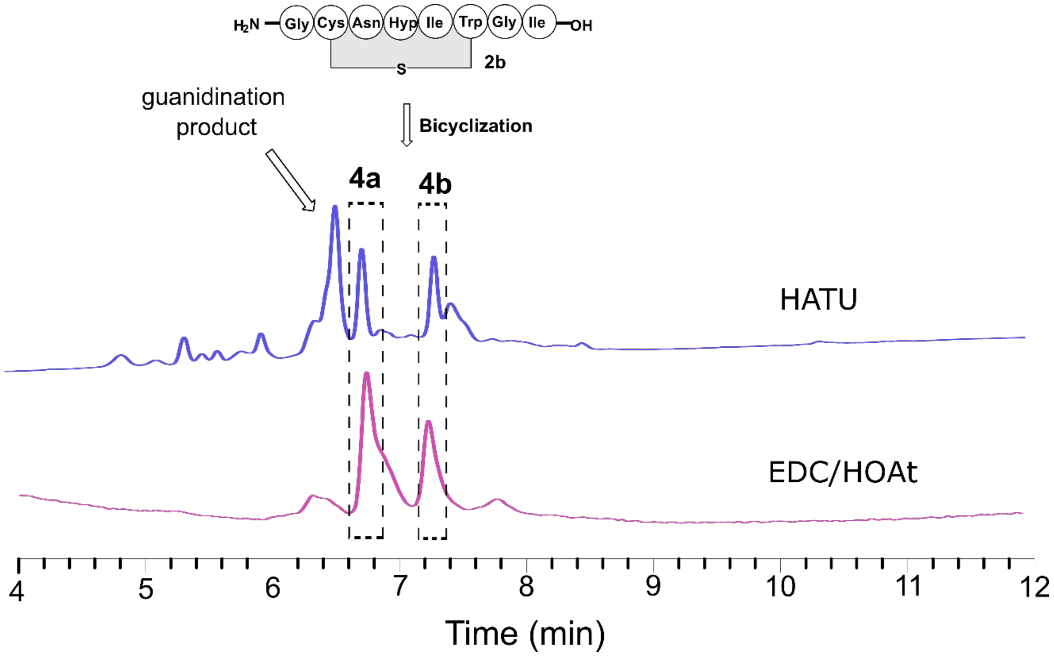

Supplementary Figure 19: Effects of coupling agents. LC-MS of macrolactamization of monocycle **2b** using HATU (blue line, top) or EDC/HOAt (pink line, bottom) as coupling reagent.

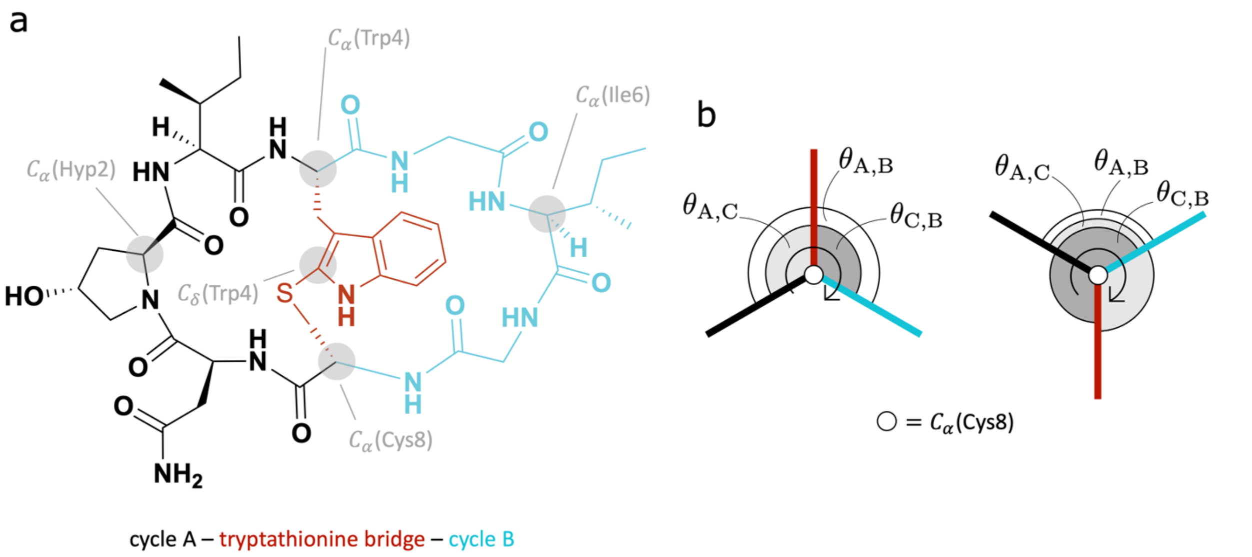

Supplementary Figure 20: Assignment of planes in the bicyclic amanitin derivatives. (a) The amanitin derivative molecule is divided into three subunits: ring A (black), ring B (cyan) and tryptathionine bridge (red). C_α atoms considered for the construction of the model shown in (b), are highlighted in gray. (b) Schematic representation of the angles between the planes E_A (black), E_B (cyan) and E_C (red). Each plane represents the subunit in (a) with the respective

color. Please note, in this scheme, the C_α atom of Trp^4 is located behind the C_α atom of Cys^8 and therefore not visible.

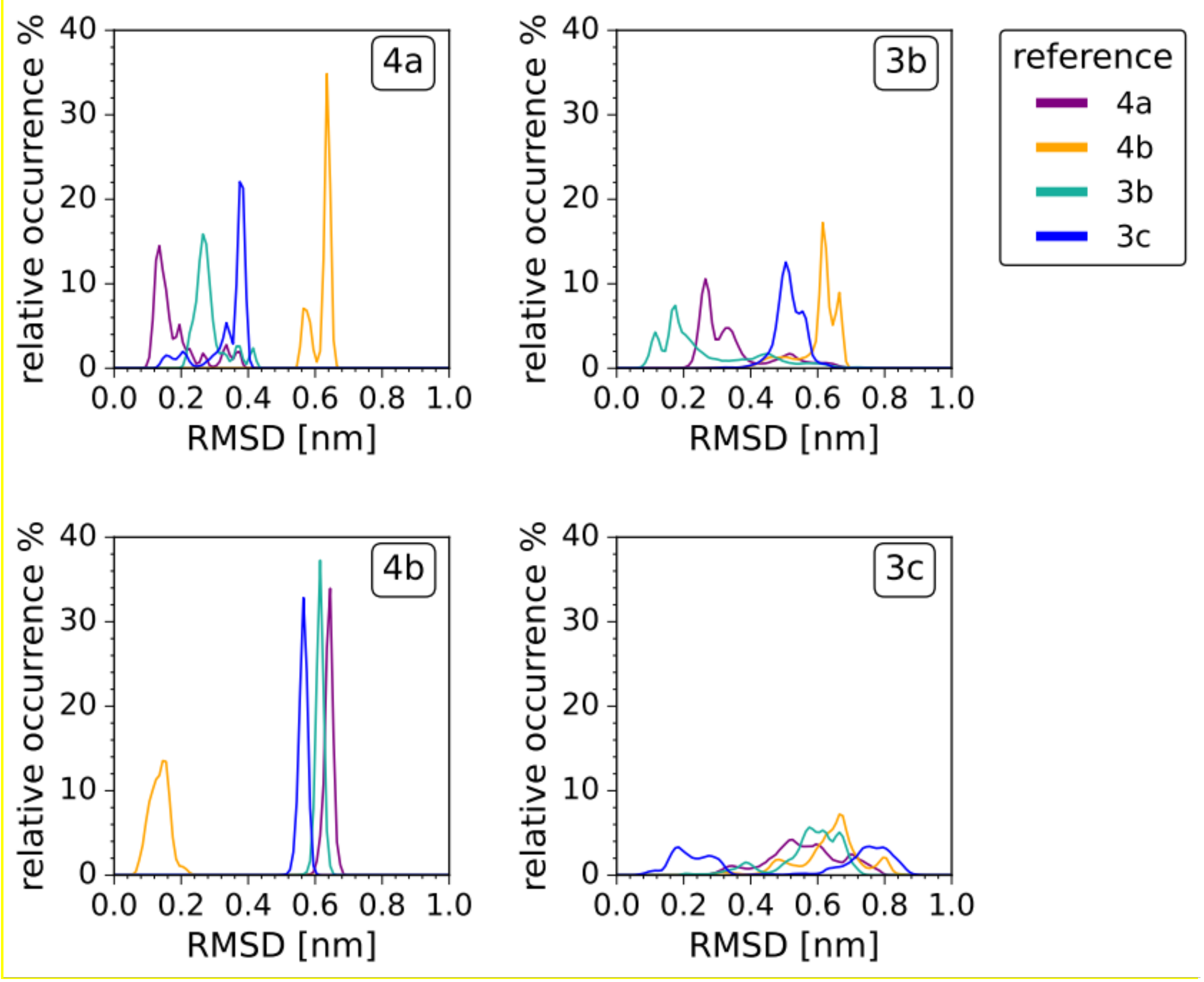

Supplementary Figure 21: **RMSD of MD structures to crystal structures.** Average distributions of the all-atom RMSD (after least-square fit on the backbone atoms of the existing ring) over all 20 replicas for each MD simulation data set (as labelled: top left: **4a**, bottom left: **4b**, top right : **3b** and bottom right : **3c**) towards different reference structures: 'purple' : **4a**, 'orange' : **4b**, 'green' : **3b**, 'blue' : **3c**. For **4a** and **4b**, the crystal structures were used as reference structures. For **3b** and **3c** the highest-probability structures were used.

2. Supplementary Tables

Supplementary Table 1: NMR shifts of **4a** and **4b**[a]

Molecule					1H Shift (ppm)								13C Shift (ppm)		
**4a**	**Residue**	HN	Hα	Hβ	Hγ	Hδ	Hε	Hε3	Hζ2	Hζ3	Hη2	Cα	Cβ	Cγ	Cδ
	1 Asn	8.52	4.67	3.38/2.94		8.23/7.50						50.04	33.08		
	2 Hyp		4.27	1.83/2.23	4.38	3.72/3.81						61.02	37.24	67.78	55.05
	3 Ile	8.04	4.18	1.93	1.46/1.16/0.89*	0.80*						57.26	34.81	24.28/15.14*	9.91
	4 Trp	8.03	4.89	3.23/3.10			11.23	7.6	7.25	7	7.11	52.82	29.13		
	5 Gly	8.08	3.38/4.25									40.43			
	6 Ile	8.5	3.68	1.56	1.56/1.10/0.78*	0.81*						58.28	33.83	24.47/14.12*	9.85
	7 Gly	8.8	3.90/3.40									41.61			
	8 Cys	8.1	4.56	2.74/3.04								51.81	37.69		
**4b**															
	1 Asn	7.31	4.96	2.52/1.89		7.27/6.71						46.23	36.54		
	2 Hyp		3.89	2.04/1.78	4	3.21/3.42						59.41	49.53	66.14	52.39
	3 Ile	7.71	3.89	1.79	1.35/0.98/0.78*	0.77*						56.33	33.01	23.63/13.85*	8.95
	4 Trp	9.04	5.68	3.27/2.96			11.48	7.54	7.3	7.05	7.14	nd	24.15		
	5 Gly	8.29	4.46/3.35									41.86			
	6 Ile	6.97	4.41	2.23	1.55/0.68/0.84*	0.79*						56.08	34.49	22.32/15.80*	10.98
	7 Gly	9.11	2.74									41.62			
	8 Cys	8.46	2.93	3.90/3.39								nd	34.13		

[a]: Note the large difference in the Cα shifts of the Asn^{1} between **4a** and **4b** and the unusual Hα and Hβ shifts for Cys^{8} in **4b**. No Cα correlations were observed in ^{1}H-^{13}C-HSQC spectra of **4b** for Trp^{4} and Cys^{8}.

Supplementary Table 2: Structure calculation data CYANA

Cycle	1	2	3	4	5	6	7	
**Peaks:**								
selected	346	346	346	346	346	346	346	
assigned	345	344	339	343	342	342	342	
unassigned	1	2	7	3	4	4	4	
with diagonal assignment	12	12	12	12	12	12	12	
**Cross peaks:**								
with off-diagonal assignment	333	332	327	331	330	330	330	
with unique assignment	196	245	258	274	285	294	291	
with short-range assignment |i-j|<=1	254	247	243	240	236	231	236	
with medium-range assignment 1<|i-j|<5	48	52	49	57	60	60	59	
with long-range assignment |i-j|>=5	31	33	35	34	34	39	35	
**Upper distance limits:**								
total	176	158	156	156	154	152	170	170
short-range, |i-j|<=1	114	100	99	95	92	87	95	95
medium-range, 1<|i-j|<5	55	51	35	40	41	41	46	46
long-range, |i-j|>=5	7	7	22	21	21	24	29	29
Average assignments/constraint	2.48	1.92	1.35	1.26	1.18	1.14	1	1
Average target function value	0.37	0.44	1.08	0.28	0.1	0.11	0.09	0.33
**RMSD (residues 1..8)**								
Average backbone RMSD to mean	0.17	0.22	0.04	0	0.02	0.07	0	0
Average heavy atom RMSD to mean	0.33	0.46	0.1	0.03	0.11	0.23	0.01	0.01

Supplementary Table 3: Approximate time points in μs for the transition from the starting structure to the crystal structure in each replica of **4a** if present. The transition times were determined as described in methods section 3.2.2.10 based on the hydrogen bond sets '1' and '2'. Hydrogen bond set '1' is the majorly-formed one and hydrogen bond set '2' represents a state that either directly (2→1) or indirectly (2→0→1) transitions into hydrogen bond set '1'.

		Hydrogen bond sets	RMSD
Replica	Transition	t [μs]	t [μs]
1	2→1	0.50	0.50
2	–	–	–
3	2→1	0.75	0.75
4	2→1	0.59	0.59
5	2→1	0.22	0.21
6	2→1	0.12	0.09
7	2→1	0.0013	–
8	2→1	0.18	–
9	2→1	0.015	–
10	2→1	0.062	0.062
11	2→1	0.26	0.27
12	–	–	–
13	2→1	0.093	–
14	2→1	0.29	0.29
15	2→1	0.30	0.30
16	2→1	0.53	0.53
17	–	–	–
18	–	–	–
19	–	–	–
20	–	–	–

Supplementary Table 4: Solvent accessible surface area (SASA) and VT-NMR data. SASA values in [nm^2] for the amides calculated as average±standard deviation over the entire simulation time (20 µs). The values were calculated using the 'shrake-rupley algorithm' available as a function of the mdtraj python library. [7] Amide proton shift changes from VT-NMR measurements are shown as ΔδHN/ΔT (ppbK^{-1}).

	**4a_MD** (nm^2)	**4a_cryst**(nm^2)	**4a_VT-NMR** ΔδHN/ΔT (ppbK^{-1})	**4b_MD** (nm^2)	**4b_cryst** (nm^2)	**4b_VT-NMR** ΔδHN/ΔT (ppbK^{-1})
**Total**	10.039 ± 0.277	10.11		9.728 ± 0.25	9.257	
Asn^1	0.006 ± 0.008	0.005	-1.550	0.006 ± 0.007	0.0	-3.826
Ile^3	0.005 ± 0.006	0.0	-1.793	0.007 ± 0.006	0.001	-6.421
Trp^4	0.006 ± 0.008	0.0	-2.910	0.033 ± 0.017	0.027	-4.807
Gly^5	0.015 ± 0.015	0.0	-1.432	0.072 ± 0.038	0.06	-4.962
Ile^6	0.039 ± 0.028	0.063	-4.171	0.003 ± 0.008	0.0	0.287
Gly^7	0.064 ± 0.03	0.131	-3.403	0.016 ± 0.025	0.036	nd
Cys^8	0.023 ± 0.024	0.031	-2.358	0.001 ± 0.005	0.0	-2.686

3. Supplementary Methods

3.1 Synthesis protocols and characterization data

3.1.1 Reagents, Solvents and Chromatographic Conditions

Commercially available reagents (Carl Roth GmbH and Co. KG, Karlsruhe, Germany; Sigma-Aldrich Taufkirchen, Germany; Iris Biotech GmbH, Marktredwitz, Germany; Orpegen, Heidelberg, Germany; ABCR, Karlsruhe, Germany; Alfa Aesar, Karlsruhe, Germany; Merck, Darmstadt; Germany; TCI, Eschborn, Germany; VWR International GmbH, Darmstadt, Germany; and Acros, Geel, Belgium) and solvents (Fisher Scientific-Acros, Schwerte, Germany) were used without further purification. If necessary, reactions were carried out under an atmosphere of argon or nitrogen and dry solvents. Analytical thin layer chromatography was carried out using aluminium-backed plates coated with silica gel (60, F254; Macherey & Nagel, Düren, Germany). Analysis was performed by visualizing the spots under UV light (λ = 254 nm), and/or by staining with $KMnO_4$ solution (3 g $KMnO_4$, 20 g K_2CO_3, 300 mL dest. H_2O, 5 mL NaOH solution (5 %)) and/or with Ninhydrin solution (0.3 g Ninhydrin, 3 mL AcOH, 100 mL *n*BuOH). Flash chromatography was carried out with silica gel (particle size 40-63 µm, VWR Chemicals, Darmstadt, Germany). Preparative HPLC was carried out on a 1260 Infinity (Agilent Technologies, Waldbronn, Germany) HPLC system with a polymeric reversed phase column (PLRP-S 100A) 300 x 50 mm, particle size 10 µm, Agilent Technologies, Waldbronn, Germany). ^1H and ^{13}C spectra were recorded at 298 K using the following spectrometers: Bruker Avance-II 400 MHz, Bruker Avance-III 500 MHz or Bruker Avance III 700 MHz (Bruker, Karlsruhe, Germany). The chemical shifts are reported in ppm using the residual solvent peak as an internal reference (DMSO-d_6, methanol-d_4, $CDCl_3$). Multiplicity (br. s = broad singlet, s = singlet, d = doublet, dd = doublet of doublet, t = triplet, q = quartet, m = multiplet) and coupling constants (J = Hz) are quoted where possible. HPLC-HRMS spectra were recorded on a QTrap LTQ XL (Thermo Fisher Scientific, Waltham, Massachusetts, USA) hyphenated to an Agilent 1200 Series HPLC-System (Agilent Technologies, Waldbronn, Germany) equipped with a C18 column (50 x 2 mm, particle size 3 µm). HPLC-HRMS chromatograms were obtained with a solvent gradient of 0.1% formic acid in water (Solvent A) and 0.1% formic acid in acetonitrile (Solvent B). The solvent gradients were either gradient A or gradient B: gradient A: 0-10 min 10%-50% B, 10-13 min 100% B, 13-16 min 20% B, gradient B: 0-10 min 20%-100% B, 10-13 min 100% B, 13-16 min 20% B Chiral HPLC was performed with a LaChrom system (Hitachi, Tokyo, Japan) equipped with a Chiralpak® Daicel-polysaccharide-column (250 x 4.6 mm, particle size 5 µm, Chiral Technologies Europe - Daicel Group, Illkirch, France).

3.1. 2 Abbreviations

CH_3CN, acetonitrile; COMU, 1-[(1-(cyan-2-ethoxy-2-oxoethylidenaminooxy)-dimethylamino-morpholino)]-uronium-hexafluorophosphate; THF, tetrahydrofuran; DCM, dichloromethane; DIPEA, *N,N'*-diisopropylethylamine; DMF, *N,N'*-dimethylformamide; TFA, trifluoroacetic acid; THF, tetrahydrofuran; HPLC, high-performance liquid chromatography; HATU, *O*-(7-azabenzotriazol-1-yl)-

N,N,N',N'-tetramethyluronium-hexafluorophosphate; 2-CTC, 2-chlorotrityl chloride (resin); TIS, triisopropylsilane; HFIP, hexafluoroisopropanol; D, L-FDLA, 1-fluoro-2,4-dinitrophenyl-5-D,L-leucine-amide; TBS, tert-butyldimethylsilyl; Fmoc, fluorenylmethoxycarbonyl protecting group. The amino acid three letter code was used for the proteinogenic amino acids according to IUPAC standards. If not otherwise stated, L-amino acids were used. Amino acid abbreviations: Hyp, *trans*-4-hydroxy-L-proline.

3.1.3 Variable temperature NMR (VT-NMR)

The temperature dependence of amide chemical shifts is well established and has been widely used to assess the solvent shielding properties of amide protons. Variable temperature NMR was carried out to assess the temperature dependency of NH chemical shifts. ^{1}H-NMR spectra were acquired from 303K to 343K (**4a**) or 300K to 354K (**4b**) in 10 K increments in DMSO-d6. Temperature coefficients are categorized as the following; $\Delta\delta HN/\Delta T$ with values less than -4.6 ppb/K indicate solvent-exposed NHs. Intermediate values from –4.6 to –3.0 ppb/K indicate intermediate shielding and potentially weak or strained hydrogen bonding. Whereas $\Delta\delta HN/\Delta T$ values greater than –3.0 ppb/K place NHs in the highly shielded and potentially strongly hydrogen bound category.

3.1.4 NMR assignment and structure calculation of desulfurized macrolactam **5**

To obtain resonance assignments for NOE assignment and structure calculations **4a**, **4b** and desulfurized macrolactam **5** were dissolved in deuterated DMSO-d_6 (approx. 10 mM). TOCSY, COSY, NOESY and ^{1}H-^{13}C-HSQC spectra were recorded on a Bruker Avance III 700 MHz spectrometer with a TXI 5 mm probe. Standard Bruker pulse programs were used and all spectra were acquired at 298 K. Residual solvent methyl peaks (DMSO-d7 δ = 2.502 for ^{1}H and δ = 39.0 ppm for ^{13}C) were used for chemical shift referencing. 2D homonuclear spectra were measured with acquisition times of 70 and 18 ms for the direct and indirect dimensions, respectively. TOCSY and NOESY spectra were accumulated with 16 or 32 (in case of **4b**) scans and COSY spectra with 8 scans. The TOCSY and NOESY mixing times were set to 100 and 300 ms, respectively. Natural abundance ^{1}H-^{13}C-HSQC spectra were measured with 140 scans and acquisition times of 14 and 120 ms for the direct and indirect dimensions. The spectra were processed and analyzed using TopSpin 3.5 (Bruker) and CcpNmr 2.3.1 [8] (The CCPN data model for NMR spectroscopy: development of a software pipeline). After shift assignment (Supplementary Table 1), the NOE correlations of **4a** and **4b** were manually assigned and residue interaction matrices of **4a** and **4b** were generated using CcpNmr.

For structure determination the manually assigned chemical shifts of the desulfurized macrolactam and NOESY peak lists were supplied to CYANA for automated NOE assignment and structure calculation (Supplementary Table 2). The program CYLIB [9] was used to generate a CYANA library file for 4-hydroxyproline. A set of 1000 structures was calculated and the 100 best were visually inspected with UCSF Chimera.[10]

3.1.5 Structure desulfurized macrolactam **5**

The calculations show a very similar backbone conformation compared to the Cys-containing macrolactam [11] with a RMSD of 0.806 Å between the two structures. Differences are largely found in the B ring around the two Gly residues and some minor differences in the side-chain orientations of Trp and Ile moieties. Comparison of J coupling constants between the two macrolactams suggests some small differences in phi angles, as observed in the calculated structures. These discrepancies could be due to the different solvents, DMF versus DMSO.

Structural alignments of **4a** and **4b** with the calculated macrolactam structure of **5** show larger differences, with the peptide ring of **4a** showing higher similarities to **5**. The RMSD between **4a** and **5** is 1.422 Å with a very similar backbone geometry of the A-ring and more differences in the B ring. The RMSD between **4b** and **5** is 2.454 Å with very different backbone geometry in rings A and B. In addition, compared to the relatively planar macrolactam, the peptide plane of **4b** shows a strong bend between rings A and B (see crystal structure and MD angle analysis of **4a**).

3.1.6 Analytical Methods

HPLC-MS: HPLC-HRMS spectra were recorded on a QTrap LTQ XL (Thermo Fisher Scientific, Waltham, Massachusetts, USA) hyphenated to an Agilent 1200 Series HPLC-System (Agilent Technologies, Waldbronn, Germany) equipped with a C18 column (50 x 2 mm, particle size 3 µm). HPLC-HRMS chromatograms were obtained with a solvent gradient of 0.1% formic acid in water (Solvent A) and 0.1% formic acid in acetonitrile (Solvent B).

The solvent gradients were shown below:

Gradient A: 0-10 min 10%-50% B, 10-13 min 100% B, 13-16 min 20% B,

Gradient B: 0-10 min 20%-100% B, 10-13 min 100% B, 13-16 min 20% B.

Gradient C: 0-10 min 50%-100% B, 10-13 min 100% B, 13-16 min 20% B.

Gradient D: 0-10 min 5%-100% B, 10-13 min 100% B, 13-16 min 20% B.

Gradient E: 0-30 min 10% to 65% B, 30-33 min 100%B, 33-36 min 20% B.

3.1.7 Experimental

3.1.7.1 General protocol

All linear peptides with different amino acid sequences were synthesized using this protocol with an alternating sequence of Fmoc-deprotections (*Method A*) and amino acid couplings (*Method B/C*).

Method A) Removal of the Fmoc group. A solution of 20% piperidine in DMF (5 mL) was added to the resin (1 g; loading 0.10-0.50 mmol/g) and the resulting suspension was shaken for 10 min. Then the solution was removed from the resin. Again, a solution of 20% piperidine in DMF (5 mL) was added

to the resin and the resulting suspension was shaken for another 10 min. The solution was drained and the resin was washed with DMF (6 x 5 mL).

Method B) Amino acid coupling. Amino acid (4.0 eq) and TBTU (4.0 eq) were dissolved in dry DMF (5 mL). DIPEA (12 eq) was added dropwise to the DMF solution. After activating for 1 min, the resulting solution was added to the Fmoc-deprotected resin (1 g; loading 0.10-0.50 mmol/g). The mixture was shaken until the coupling reaction was completed. Then, the solution was drained and the resin was rinsed with DMF (4 x 5 mL).

Method C) Amino acid coupling. Amino acid (4.0 eq) and HATU (4.0 eq) were dissolved in dry DMF (5 mL). DIPEA (12 eq) was added dropwise to the DMF solution. After activating for 1 min, the resulting solution was added to the Fmoc-deprotected resin (1 g; loading 0.10-0.50 mmol/g). The mixture was shaken until the coupling reaction was completed. Then, the solution was drained and the resin was rinsed with DMF (4 x 5 mL).

3.1.7.2 I_2-mediated thioether formation

The thioether bridge (tryptathionine motif) was obtained from linear resin-bound peptide (1 eq; 500 mg; loading 0.10-0.50 mmol/g) synthesized according to the above method. Formation of the thioether was achieved by adding a freshly prepared solution of iodine in DMF (2 eq, 2 mg/ml) under protecting gas atmosphere (Ar or nitrogen). The mixture was shaken under nitrogen atmosphere for 2.5 h to complete the formation of the thioether. If required also longer reaction times were applied (HPLC-MS control of a test cleavage). Then, the solution was drained and the resin was rinsed with DMF (4 x 3 mL).

3.1.7.3 Cleavage from solid support

Condition A) The resin (1 g; loading 0.3 mmol/g) was treated with 10 mL of a mixture of TFA/TIS/H2O (95:2.5:2.5) for 1 h at room temperature with gentle agitation. The resin was filtered and rinsed with 1% TFA in DCM (2 x5 mL). The rinses and filtrate were combined and evaporated to dryness.

Condition B) The resin (1 g; loading 0.10-0.50 mmol/g) was treated with 10 mL of a mixture of HFIP/DCM (3:7) for 2 h at room temperature with gentle agitation. The resin was filtered and rinsed with DCM (2 x5 mL). The rinses and filtrate were combined and evaporated to dryness.

3.1.7.4 Monitoring of Peptide Coupling and Capping

Chloranil test: During the coupling reaction, a few resin beads were taken out and rinsed with DMF (2 x 1 mL). To the resin were added 2 drops of a 2% solution of acetaldehyde and 2 drops of a 2% solution of chloranil in DMF. The resulting suspension was allowed to stand for 5 min at room temperature. Blue- to green-stained beads indicated the presence of secondary amines.

Kaiser Test: During the coupling reaction, a few resin beads were taken out and rinsed with DMF (2 x 1 mL). To the resin were added 2-3 drops of reagent A (16.5 mg of KCN dissolved in 25 mL of distilled water. 1.0 mL of above solution diluted with 49 mL of pyridine), 2 to 3 drops of reagent B (1.0 g of ninhydrin dissolved in 20 mL of n-butanol.) and 2 to 3 drops of reagent C (40 g of phenol dissolved

in 20 mL of n-butanol). The resulting suspension was allowed to heat at 110°C for 3 min, blue- to green-stained beads indicated the presence of primary amine.

3.1.7.5 Macrolactamization

Condition A) To a solution of monocyclic peptide in solution of DPEA (5 eq) in DMF, HATU (2 eq) was added at 0°C. The solution was stirred for 12 h, followed by preparative HPLC purification. The isolated product was lyophilized to give the white solid.

Condition B) To a solution of EDCI (2 eq) and HOAt (2 eq) in solution of DPEA (5 eq) in DMF, monocyclic peptide was added at 0°C. The solution was stirred for 12 h, followed by preparative HPLC purification. The isolated product was lyophilized to give the white solid.

3.1.7.6 Deprotection

Detritylation: The final protected bicyclic peptides were dissolved in DCM (2 ml) and followed by addition of TFA/TIS/H_2O (95:2.5:2.5, 2ml) and were stirred for 30 min. Afterwards, the reaction mixture was evaporated under reduced pressure and the followed crude product was purified by preparative HPLC to give the final compound.

3.1.8 Synthesis and characterization data

3.1.8.1 Synthesis of monocyclic peptides **2a-2d** and **3a-3d**

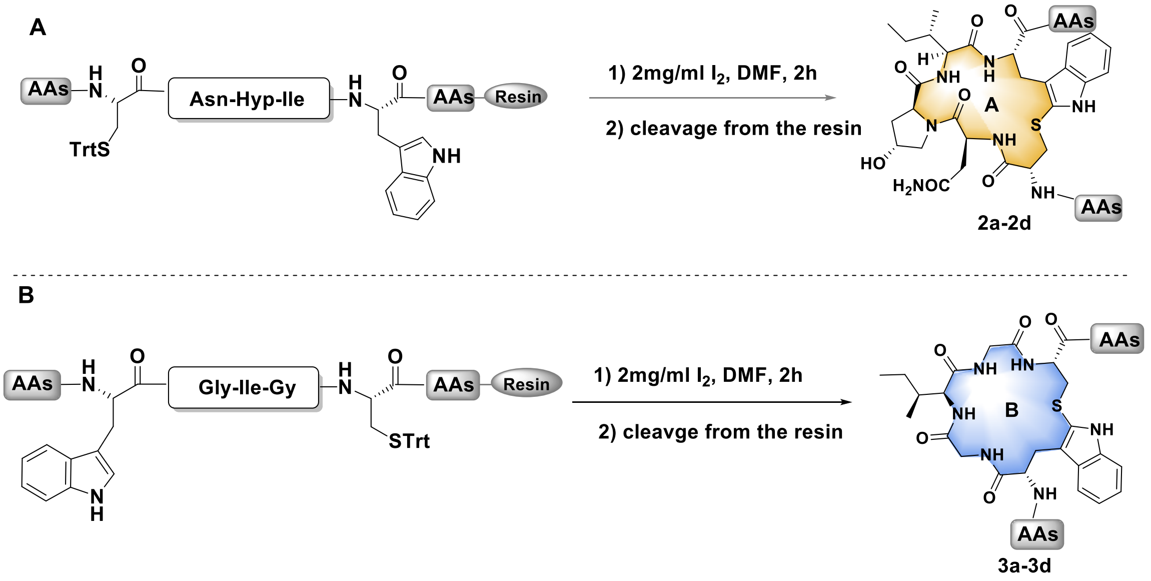

Supplementary Figure 22. Synthesis scheme of octapeptides. Synthesis of monocyclic octapeptides (**2a-2d** and **3a-3d**) with tryptathionine bridge. A) Synthesis scheme for formation of monocycle A (yellow) in peptides **2a-2d**. B) Synthesis scheme for formation of monocycle B (blue) in peptides **3a**-3d.

General protocol for solid-phase peptide synthesis (SPPS):

2-CTC resin (1 g, 0.98 mmol/g) was pre-swollen for 20 min in DCM in a manual solid phase peptide synthesis vessel (10 mL). After the solvent was drained, the first amino acid Fmoc-AA^1-OH (0.3 mmol) and DIPEA (0.26 mL, 1.5 mmol) in DCM (5 mL) were added to the resin. The mixture was agitated for 2 hbefore the solvent was drained. The resin was rinsed with DMF (4 x 3 mL). Then a mixture of MeOH/DIPEA/DCM (1:1:8) was added to cap the remaining 2-chlorotrityl chloride on the resin. The mixture was agitated for 0.5 h. Then the solvent was drained and the resin was washed with DMF (4 x 3 mL). The resin loading was determined to be 0.30 mmol/g. The Fmoc-group was removed according to Method *A*. Fmoc-AA^2-OH (4 eq) was coupled to the deprotected resin according to Method *B*. The Fmoc-group of the resulting resin was removed according to Method *A*. The following six amino acids were coupled to the deprotected resin according to Method *A* and *B*. The tryptathionine formation was carried out on the solid support. After removal of Fmoc-group using method *A* and followed cleavage from the resin, the monocyclic peptide was obtained following subsequent HPLC purification. The synthesis and characterization data of **2a-2d**, **3a-3b** and **3d** have been reported in our previous paper.[11]

2a: **H$_2$N-Gly-Ile-Gly-(*cyclo-tryptathionine*)[Cys-Asn-Hyp-Ile-Trp]-OH**

HRMS (ESI): m/z calculated: $C_{39}H_{57}N_{10}O_{11}S^+$ [M+H]$^+$ 873.3923, found 873.3912.

HPLC-MS: Retention time R_t = 8.08 min (Gradient B).

2b: **H$_2$N-Ile-Gly-(*cyclo-tryptathionine*)[Cys-Asn-Hyp-Ile-Trp]-Gly-OH**

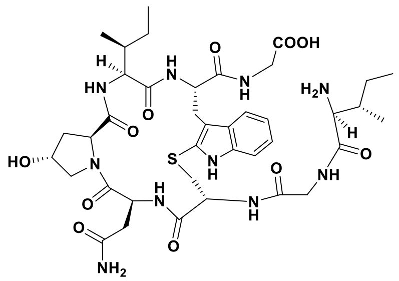

Mixed conformers: ^{1}H NMR (400 MHz, DMSO-*d*$_6$) δ 12.3 (s, 1.65H), 11.19 (s, 1H), 11.00 (s, 0.65 H), 8.69 (t, *J* = 5.6 Hz, 1 H), 8.60 (t, *J* = 5.6 Hz, 1H), 8.44 (d, *J* = 8.6 Hz, 0.65 H), 8.32 (d, *J* = 7.9 Hz, 0.65 H), 8.25 (t, *J* = 5.8 Hz, 0.65 H), 8.11 (s, 5H), 7.87 (t, *J* = 9.5 Hz, 2H), 7.70 (s, 1H), 7.60 (d, *J* = 7.9 Hz, 0.65 H), 7.57 (s, 1H), 7.47 (d, *J* = 8.0 Hz, 1H), 7.34 (s, 1H), 7.27 (t, *J* = 8.5 Hz, 2H), 7.17 – 6.92 (m, 4H), 6.83 (s, 0.65 H), 4.77 – 4.53 (m, 5H), 4.32 – 4.25 (m, 2H), 4.16 – 4.11 (m, 1H), 4.05 (dd, *J* = 16.7, 5.8 Hz, 1H), 3.97-3.88 (m, 3H), 3.87 – 3.78 (m, 3H), 3.75 – 3.57 (m, 3H), 3.48 (d, *J* = 11.1 Hz, 1H), 3.43 – 3.27 (m, 1H), 3.22 – 3.08 (m, 5H), 3.04 – 2.97 (m, 1H), 2.92 (d, *J* = 9.8 Hz, 1H), 2.71 – 2.58 (m, 3H), 2.26 (dd, *J* = 15.4, 4.0 Hz, 1H), 2.16 – 1.97 (m, 3H), 1.88 – 1.65 (m, 2H), 1.59 – 1.30 (m, 3H), 1.26 – 1.10 (m, 1.5H), 1.06– 0.97 (m, 1H), 0.96 – 0.82 (m, 10H), 0.77 (t, *J* = 7.3 Hz, 2H), 0.73 (d, *J* = 6.8 Hz, 2H), 0.60 (s, 3H), 0.39 (s, 3H).

^{13}C NMR (101 MHz, DMSO-d_6_HSQC) δ 123.3, 122.6, 122.3, 119.6, 119.4, 119.0, 119.2, 111.3, 69.3, 67.4, 61.7, 60.0, 59.8, 58.0, 56.8, 55.4, 54.0, 53.0, 50.8, 47.4, 41.9, 41.4, 41.2, 37.1, 34.3, 28.0, 27.8.

HRMS (ESI): m/z calculated: $C_{39}H_{57}N_{10}O_{11}S^+$ $[M+H]^+$ 873.3923, found 873.3917.

HPLC-MS: Retention time R_t = 7.40 min (Gradient B).

2c: **H_2N-Gly-(*cyclo-tryptathionine*)[Cys-Asn-Hyp-Ile-Trp]-Gly-Ile-OH**

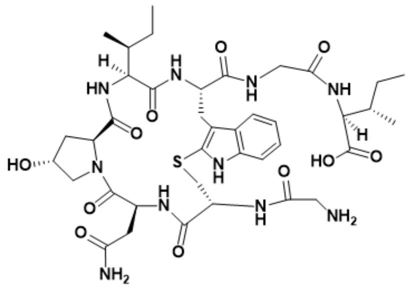

Major conformer: 1H NMR (500 MHz, DMSO-d_6) δ 12.49 (s, 1H), 11.17 (s, 1H), 8.86 (s, 1H), 8.35 (d, J = 7.8 Hz, 1H), 8.07 (t, J = 5.9 Hz, 1H), 8.00 (d, J = 6.0 Hz, 1H), 7.72 (d, J = 6.3 Hz, 1H), 7.51 (s, 1H), 7.48 (d, J = 7.8 Hz, 1H), 7.44 (d, J = 8.0 Hz, 1H), 7.26 (d, J = 8.3 Hz, 1H), 7.12 – 7.08 (m, 1H), 6.99 (t, J = 7.4 Hz, 1H), 4.38 (d, J = 3.9 Hz, 1H), 4.30 – 4.21 (m, 1H), 4.07 (t, J = 7.2 Hz, 1H), 3.82 – 3.73 (m, 2H), 3.21 (dt, J = 14.7, 7.7 Hz, 1H), 3.15 (dd, J = 15.2, 5.5 Hz, 1H), 2.68 – 2.59 (m, 1H), 2.13 – 2.03 (m, 1H), 1.88 – 1.78 (m, 2H), 1.43-1,32 (m, 2H), 1.24-1,15 (m, 1H), 1.04 (s, 1H), 0.89 – 0.83 (m, 10H), 0.81-0.72 (m, 3H).

^{13}C NMR (126 MHz, DMSO-d_6_HSQC) δ 122.5, 119.6, 119.4, 119.2, 111.4, 69.4, 67.4, 62.1, 59.8, 57.9, 57.1, 56.6, 55.7, 53.5, 42.9, 42.7, 40.7, 40.5, 40.3, 39.8, 37.6, 36.7, 36.2, 34.6, 28.0, 25.3, 25.1, 24.9, 15.7, 15.5, 11.6, 11.2.

HRMS (ESI): m/z calculated: $C_{39}H_{57}N_{10}O_{11}S^+$ $[M+H]^+$ 873.3923, found 873.3915.

HPLC-MS: Retention time R_t = 8.67 min (Gradient B).

2d:**H_2N-(*cyclo-tryptathionine*)[Cys-Asn-Hyp-Ile-Trp]-Gly-Ile-Gly-OH**

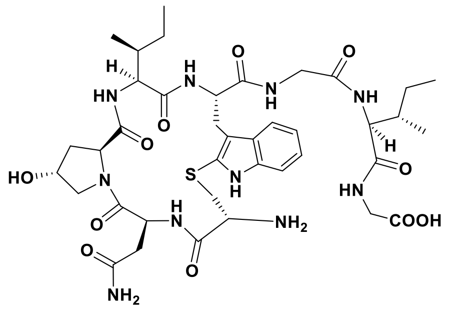

Mixed conformers: ^{1}H NMR (500 MHz, DMSO-d_6) δ 12.47 (s, 1H), 11.29 (s, 0.55 H), 11.15 (s, 0.45H), 8.63 (d, *J* = 8.1 Hz, 0.45 H), 8.38 – 8.32 (m, 3H), 8.15 (s, 0.45 H), 8.03 – 7.95 (m, 1H), 7.93 (d, *J* = 8.9 Hz, 1H), 7.79 (d, *J* = 11.6 Hz, 1H), 7.63 (d, *J* = 8.0 Hz, 0.55 H), 7.50 – 7.29 (m, 3H), 7.15 (t, *J* = 6.9 Hz, 1H), 7.02 (t, *J* = 6.9 Hz, 1H), 6.82 (s, 0.45 H), 4.80 (d, *J* = 3.2 Hz, 0.55 H), 4.74 (dd, *J* = 8.3, 6.3 Hz, 0.45 H), 4.64 – 4.50 (m, 1H), 4.41 (t, *J* = 3.7 Hz, 0.55 H), 4.34 – 4.21 (m, 1H), 4.14 – 4.07 (m, 1H), 3.96 – 3.83 (m, 1H), 3.83 – 3.52 (m, 3H), 3.38 – 3.30 (m, 1H), 3.24 – 3.17 (m, 1H), 3.15 – 3.07 (m, 1H), 2.96 (d, *J* = 6.2 Hz, 1H), 2.78-2.63 (m, 1H), 2.29 – 2.22 (m, 0.55 H), 2.17 – 2.10 (m, 1H), 1.91– 1.81 (m, 2H), 1.76 – 1.66 (m, 1.5H), 1.57 – 1.39 (m, 2.5 H), 1.20 – 1.05 (m, 2.5H), 0.92 – 0.77 (m, 14H).

^{13}C NMR (126 MHz, DMSO-d_6_HSQC) δ 123.0, 119.9, 119.6, 119.4, 111.5, 69.6, 69.2, 67.5, 67.4, 62.6, 59.5, 59.3, 59.1, 58.6, 58.5, 57.8, 56.9, 56.4, 55.9, 55.7, 54.7, 53.5, 53.3, 53.0, 51.4, 49.9, 47.7, 47.5, 43.4, 42.4, 42.2, 41.0, 40.8, 40.7, 40.0, 38.9, 38.8, 37.7, 37.6, 37.2, 36.4, 35.5, 34.8, 34.7, 28.5, 28.3, 26.8, 25.8, 25.6, 24.6, 24.4, 24.2, 16.3, 15.8, 15.7, 15.5, 13.3, 12.2, 11.6, 11.4, 11.0.

HRMS (ESI): m/z calculated: $C_{39}H_{57}N_{10}O_{11}S^+$ $[M+H]^+$ 873.3923, found 873.3913.

HPLC-MS: Retention time R_t = 8.42 min (Gradient B).

3c: H_2N-Hyp-Ile-(*cyclo-tryptathionine*)[Trp-Gly-Ile-Gly-Cys]-Asn(Trt)-OH

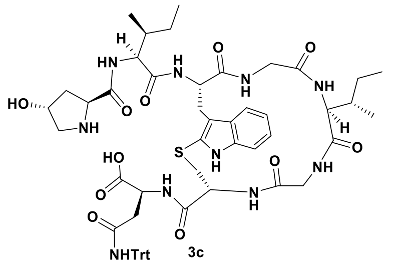

After loading the Fmoc-Asn(Trt)-OH (0.3 mmol) on the resin according the general synthesis, the following peptide sequences Fmoc-Cys(Trt)-OH, Fmoc-Gly-OH (4 eq), Fmoc-L-Ile-OH (4 eq), Fmoc-Gly-OH (4 eq), Fmoc-L-Trp-OH (4 eq), Fmoc-L-Ile-OH (4 eq) and Fmoc-Hyp-OH (4 eq) were coupled to the deprotected resin according to Method *A* and *B*. The tryptathionine formation was carried out on the solid support. After removal of Fmoc group using method *A* and followed cleavage from the resin using cleavage condition B, 150 mg peptide **3c** was obtained as white solid powder with 55% yield following subsequent HPLC purification.

^{1}H NMR (500 MHz, DMSO-d_6) δ 12.61 (s, 1H), 11.23 (s, 1H), 9.53 (s, 1H), 8.61 (s, 1H), 8.58 (s, 1jH), 8.53 (d, *J* = 8.4 Hz, 1H), 8.29 – 8.16 (m, 2H), 8.19 (d, *J* = 7.6 Hz, 1H), 8.13 (d, *J* = 7.7 Hz, 1H), 7.76 (t, *J* = 4.7 Hz, 1H), 7.68 (d, *J* = 5.8 Hz, 1H), 7.65 (t, *J* = 5.5 Hz, 1H), 7.31 – 7.18 (m, 7H), 7.21 – 7.07 (m, 9H), 7.01 – 6.95 (t, *J* = 7.6 Hz, 1H), 5.46 (s, 1H), 4.69 (d, *J* = 2.5 Hz, 1H), 4.53 – 4.40 (m, 2H), 4.33 (d, *J* = 4.5 Hz, 1H), 4.27 – 4.18 (m, 1H), 4.08 (t, *J* = 8.0 Hz, 1H), 4.04 (t, *J* = 7.4 Hz, 1H), 3.92 (dd, *J* = 15.3, 5.9 Hz, 1H), 3.87 (d, *J* = 5.8 Hz, 1H), 3.72 (dd, *J* = 16.8, 4.4 Hz, 1H), 3.65 (dd, *J* = 15.5, 4.7 Hz, 1H), 3.39 (dd, *J* = 13.5, 3.6 Hz, 1H), 3.32 – 3.26 (m, 2H), 2.95 – 2.90 (m, 1H), 2.89 – 2.82 (m, 1H), 2.79 – 2.73 (m, 1H), 2.56 (dd, *J* =

15.6, 7.0 Hz, 1H), 2.14 (dd, J = 13.3, 7.3 Hz, 1H), 1.93 (qd, J = 6.7, 3.4 Hz, 1H), 1.60 (dd, J = 10.2, 3.9 Hz, 1H), 1.30 – 1.22 (m, 1H), 1.20 – 1.10 (m, 1H), 0.99 (m, 1H), 0.91 (d, J = 8.8 Hz, 0H), 0.88 (d, J = 6.8 Hz, 3H), 0.83 (t, J = 7.4 Hz, 3H), 0.76 (t, J = 7.4 Hz, 3H), 0.65 (d, J = 6.7 Hz, 3H).

^{13}C NMR (101 MHz, DMSO-d_6_HSQC) δ 129.0, 127.9, 126.9, 122.5, 119.5, 119.0, 111.3, 69.3, 59.2, 58.8, 58.0, 57.8, 53.9, 53.5, 52.0, 49.2, 43.2, 42.7, 38.8, 38.1, 37.9, 36.7, 35.7, 35.5, 29.0, 28.7, 28.4, 24.6, 24.3, 24.1, 15.7, 15.2, 11.3, 11.1.

HRMS (ESI): m/z calculated: $C_{58}H_{71}N_{10}O_{11}S^+$ $[M+H]^+$ 1115.5019, found.1115.5009

HPLC-MS: Retention time R_t= 6:98 min (Gradient B)

3.1.8.2 Synthesis of bicyclic peptides **4a** and **4b**

General synthesis: Monocyclic octapeptide **2a-2d** and **3a-3d** (1.0 eq) was dissolved in DMF (1 mM). Then, DIPEA (2.2 eq) and HATU (2.0 eq) was added at 0°C. The reaction mixture was allowed to warm to r.t. for 12 h and concentrated under reduced pressure. The crude product was purified using preparative HPLC to afford bicyclic octapeptide as a white powder. Since large amounts of guanidination product were detected during macrolactamization of **2b** (see supplementary figure 19), the alternative coupling condition EDC (2 eq) and HOAt (2 eq) was employed to cyclize the monocyclic peptide **2b**. All results of LC-MS runs are shown in Supplementary Figure 1. In addition, the detritylation was performed after macrolactamization of **3c**. The yield and ratio of **4a** and **4b** is shown in the table below and in Figure 2.

Natural ansamer: Ile^3-*S*-deoxo-amaninamide (**4a**)

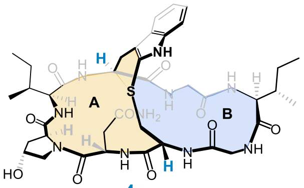

4a

^{1}H NMR (500 MHz, DMSO-d_6) δ 11.22 (s, 1H), 8.80 (dd, J = 7.4, 5.1 Hz, 1H), 8.50 (dd, J = 13.2, 3.7 Hz, 2H), 8.22 (s, 1H), 8.09 (t, J = 8.7 Hz, 2H), 8.04 (t, J = 9.0 Hz, 2H), 7.60 (d, J = 8.0 Hz, 1H), 7.49 (s, 1H), 7.25 (d, J = 8.1 Hz, 1H), 7.11 (t, J = 7.4 Hz,1H), 7.00 (t, J = 7.3 Hz, 1H), 5.75 (s, 1H), 4.89 (dt, J = 13.3, 6.8 Hz, 1H), 4.66 (q, J = 4.0 Hz, 1H), 4.56 (ddd, J = 12.5, 10.1, 3.7 Hz, 1H), 4.31 – 4.24 (m, 2H), 4.25 – 4.14 (m, 2H), 3.90 (dd, J = 17.3, 7.5 Hz, 1H), 3.84 – 3.78 (m, 1H), 3.68 (dd, J = 8.3, 4.1 Hz, 1H), 3.39 (ddd, J = 17.2, 8.6, 4.8 Hz, 3H), 3.23 (dd, J = 14.9, 13.0 Hz, 1H), 3.14 – 3.00 (m, 2H), 2.94 (dd, J = 15.7, 4.3 Hz, 1H), 2.74 (dd, J = 10.8, 3.6 Hz, 1H), 2.23 (dd, J = 12.9, 7.0 Hz, 1H), 1.98 – 1.88 (m, 1H), 1.83 (td, J = 12.4, 3.5 Hz, 1H), 1.61 – 1.43 (m, 2H), 1.22 – 1.04 (m, 2H), 0.89 (d, J = 6.8 Hz, 3H), 0.85 – 0.75 (m, 10H).

^{13}C NMR (126 MHz, DMSO-d_6_HSQC) δ 122.8, 120.9, 119.0, 111.7, 68.9, 62.1, 59.3, 59.0, 58.3, 55.9, 53.7, 53.0, 51.1, 42.5, 41.9, 41.5, 38.6, 38.1, 36.0, 35.9, 34.7, 34.2, 34.0, 33.8, 30.0, 29.9, 25.3, 25.1, 15.8, 14.8, 12.2, 10.7

HRMS (ESI): m/z calculated: $C_{39}H_{55}N_{10}O_{10}S^+$, 855.3818, found 855.3843

HPLC-MS: Retention time R_t = 5.73 min (Gradient B)

Non-natural atropisomer: Ile^3-*S*-deoxo-amaninamide (**4b**)

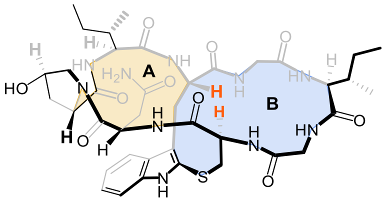

4b

^{1}H NMR (500 MHz, DMSO-d_6) δ 11.48 (s, 1H), 9.05 (s, 1H), 8.46 (s, 1H), 8.28 (s, 3H), 7.71 (s, 1H), 7.54 (s, 1H), 7.33 – 7.26 (m, 2H), 7.14 (dd, *J* = 8.1, 6.9 Hz, 1H), 7.13 – 6.97 (m, 2H), 6.71 (s, 1H), 5.67 (s, 1H), 4.97 (td, *J* = 9.8, 3.0 Hz, 1H), 4.75 (m, 2H), 4.48 (dd, *J* = 16.8, 9.7 Hz, 2H), 4.42 (dd, *J* = 10.2, 3.3 Hz, 1H), 4.01 (s, 1H), 3.95 – 3.88 (m, 3H), 3.49 – 3.40 (m, 1H), 3.39 – 3.20 (m, 2H), 2.98 (d, *J* = 15.2 Hz, 2H), 2.24 (s, 1H), 1.91 (d, *J* = 15.2 Hz, 1H), 1.79 (s, 2H), 1.56 (s, 1H), 1.36 (s, 1H), 1.03 – 0.95 (m, 1H), 0.85 (d, *J* = 6.7Hz, 3H), 0.81-0.76 (m, 8H).

^{13}C NMR (176 MHz, DMSO-d_6_HMBC) δ 137.2, 128.4, 128.2, 119.4, 118.7, 111.1, 67.0, 57.3, 57.1, 47.1, 37.6, 35.4, 34.2, 31.7, 23.5, 14.7.

HRMS (ESI): m/z calculated: $C_{39}H_{55}N_{10}O_{10}S^+$, 855.3818, found 855.3837

HPLC-MS: Retention time R_t = 6.47 min (Gradient B)

3.1.8.3 Amino acid analysis of **4a** and **4b** (Marfey's reagent)

The absolute configurations of the amino acid units in **4a** and **4b** were determined by the advanced Marfey's method. The hydrolysis products (6 N HCl, 110 °C, 12 h) of **4a** and **4b** were subjected to L-FDLA and L,D-FDLA derivatization and analyzed by LC-MS, which showed that amino acid residues of **4a** were identical with **4b** (see Supplementary Figure 4). Protocol: Isomers **4a** and **4b** (0.1 mg each) were hydrolyzed with stirring in 6 M HCl (200 µL) at 110 °C for 12 h. The residual HCl fumes were removed under a N_2 stream. Acid hydrolysates (suspended in 50 µL of H_2O) were treated with 1 M $NaHCO_3$ (20 µL) and then with L-FDLA and L,D-FDLA (100 µL of a 10 mg/mL solution in acetone), and the mixture was stirred at 37 °C for 1 h. The reaction was quenched with 1 M HCl (20 µL) and then diluted with MeOH for subsequent analysis.

3.1.8.4 Heating experiment of **4a** and **4b**

To investigate the interconversion of **4a** and **4a**, the heating experiment was performed (see Supplementary Figure 17). Protocol: After replacing the air with Argon, the NMR samples of **4a** and **4b** were heated to 150 °C for 10h. The proton NMR spectra were acquired after cooling down to room temperature.

3.1.8.5 Desulfurization of **4a** and **4b**

4a and **4b**

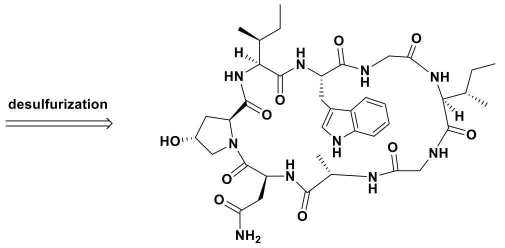

Raney nickel solution in water was added to the bicyclic peptide **4a** or **4b** dissolved in MeOH, which was then immediately sealed. The reaction was heated to 80 °C for 3h. The extent of desulfurization was monitored by LC-MS, the monocyclic product was obtained following HPLC purification. The results showed that identical product was obtained after desulfurization.

^{1}H NMR (700 MHz, DMSO-d_6) δ 10.71 (d, J = 2.4 Hz, 1H), 9.12 (t, J = 5.2 Hz, 1H), 8.62 (d, J = 8.3 Hz, 1H), 8.07 (s, 1H), 7.92 (t, J = 6.2 Hz, 1H), 7.66 (d, J = 7.9 Hz, 1H), 7.46 (d, J = 7.9 Hz, 1H), 7.42 (s, 1H), 7.35 (d, J = 8.0 Hz, 1H), 7.09 (d, J = 2.6 Hz, 1H), 7.07 (t, J = 7.4 Hz, 2H), 7.01 (t, J = 7.4 Hz, 1H), 6.95 (d, J = 8.9 Hz, 1H), 4.95 (t, J = 3.2 Hz, 1H), 4.45 – 4.39 (m, 2H), 4.38 – 4.33 (m, 1H), 4.35 – 4.29 (m, 1H), 4.24 (t, J = 7.6 Hz, 1H), 3.97 (dd, J = 10.8, 3.7 Hz, 1H), 3.90 – 3.84 (m, 1H), 3.87 – 3.81 (m, 1H), 3.70 – 3.67 (m, 1H), 3.65 (dd, J = 14.7, 4.2 Hz, 1H), 3.49 (dd, J = 16.4, 5.6 Hz, 1H), 3.47 – 3.44 (m, 1H), 3.28 – 3.22 (m, 2H), 2.67 (dd, J = 14.9, 10.3 Hz, 1H), 2.55 – 2.52 (m, 1H), 2.12 (d, J = 4.7 Hz, 1H), 2.00 – 1.87 (m, 2H), 1.58 – 1.53 (m, 1H), 1.47-1.37 (m, 2H), 1.30 (d, J = 7.2 Hz, 3H), 1.22 – 1.13 (m, 1H), 1.00 – 0.91 (m, 1H), 0.81-0.75 (m, 10H), 0.56 (d, J = 6.9 Hz, 3H).

^{13}C NMR (101 MHz, DMSO-d_6_HSQC) δ 123.5, 121.6, 121.4, 119.2, 118.9, 118.0, 117.8, 112.0, 111.8, 68.4, 61.7, 60.2, 59.8, 55.4, 55.2, 54.4, 48.2, 47.5, 44.3, 44.1, 43.7, 43.6, 39.1, 38.8, 38.4, 37.8, 34.0, 28.5, 28.2, 25.9, 24.2, 23.9, 18.3, 16.7, 16.5, 15.4, 15.2, 13.3, 13.1, 11.3, 11.1, 10.2.

HRMS (ESI): m/z calculated: $C_{39}H_{57}N_{10}O_{10}^{+}$, 825.4257, found 825.4258

HPLC-MS: Retention time R_t = 5.60 min (Gradient B)

3.1.9 CD spectroscopy

The structures of the isomers were evaluated using CD spectroscopy. The measurements were conducted on a J-815 CD spectrometer (Jasco, Groß-Umstadt, Germany). Lyophilized compounds **4a** and **4b** were dissolved in H_2O to reach a concentration of 75 µM. Far-UV spectra were acquired at 20°C 190-300 nm with a path length of 0.1 cm and a bandwidth of 1 nm at a continuous scanning speed of 50 nm/min

in 5 accumulations. The data pitch was set to 0.1 nm. The spectra were processed with Spectra Manager (JASCO) and the mean residue ellipticity (MRE) was calculated

$$MRE = \frac{\theta * 0.1}{l * c * n} \qquad (1)$$

Where θ is the ellipticity and l, c, and n denote the path length, molar concentration and number of amino acids.

3.2 Molecular dynamics simulations

We performed classical, all-atom molecular dynamics simulations for the conformational isomers **4a** and **4b** as well as the precursor **3b**. See table below for the summary of the simulations:

Supplementary Table 5. Overview of the MD simulations for the molecules **3b**, **4a** and **4b** in the named solvent with temperature T [K], number of replicas and total simulation time t_{sim} [µs]. For the convenience of the reader, a short description of each molecule is given.

Mol.	Description	Solvent	T [K]	Replicas	t_{sim} [µs]
4a	Derivative of the natural amanitin; tryptathionine bridge above of macrolactam	DMF	300<br>400	20<br>1	20<br>0.1
4b	Conformational isomer of **4a** with the tryptathionine bridge below the macrolactam	DMF	300<br>400	20<br>1	20<br>0.1
3b	Precursor with the cut between Hyp[2] and Ile[3]; N-terminus: Ile[3], C-terminus: Hyp[2]	DMF	300<br>400	20<br>1	20<br>0.1
3c	Precursor with the cut between Hyp[2] and Asn[1]; N-terminus: Hyp[2], C-terminus: Asn[1]<br>Protection group on Asn[1]: Trt	DMF	300<br>400	20<br>1	20<br>0.1

3.2.1 Setup of the MD simulations

3.2.1.1 Parametrization of the peptides **4a**, **4b**, **3b** and **3c**

MD simulations for the peptides **4a**, **4b**, **3b** and **3c** were performed with GROMACS 2019.4 [12] simulation package in dimethylformamide (DMF). Both solvent and solute were built with the molecule editor Avogadro [13] and parametrized with ACPYPE [14] setting the molecule's charge n=0 and referring to AMBER14SB[15] force field with a='amber'. The C- and N-terminus in the precursors **3b** and **3c** were capped with methyl-groups, yielding -CO-OMe and -NH-Me as end groups.

3.2.1.2 Parameters for MD simulation of the peptides **4a**, **4b**, **3b** and **3c**

After their construction with Avogadro [13] and parametrization with ACPYPE [14], the structures of the peptides were solvated in a cubic simulation box (average volume 50 nm^3) using the GROMACS commands 'gmx editconf', 'gmx solvate' and the parametrized DMF molecule as template. The distance of the solute to the box walls was set to 1 nm. On average, 389 solvent molecules were added.

The solvated systems were energy-minimized using the steepest-descent algorithm (emtol=1000 kJ/(mol$\cdot$ nm), nsteps=5000), followed by NVT and NpT equilibrations at temperature T=400 K for 400 ps and 600 ps,

respectively. In both equilibration steps, periodic boundary conditions were applied in all directions and position restraints were used for the peptides.

As pre-production run, the systems were propagated using leap-frog integration [16] with an integration time step dt = 2 fs. All covalent bonds were constrained using LINCS algorithm [17] ('all-bonds', iter = 4, order = 6). Periodic boundary conditions were applied in all three directions. The simulations were conducted in the *NpT* ensemble at temperature T=400 K (velocity-rescale thermostat [18], coupling time τ_T = 0.1 ps) and with pressure p=1 bar (Parrinello-Rahman barostat [19], coupling time τ_p = 2 ps). For Van-der-Waals interactions, Verlet-cutoff scheme [20] was applied using a cut-off radius of r_{vdw}=1 nm and updating the neighborlist every nstlist = 10 integration timesteps. For Coulomb interactions, Particle-Mesh-Ewald algorithm [21] (pme-order = 6, Fourier grid spacing = 0.12 nm, cut-off for short-range electrostatic interaction r_{Coulomb} = 1 nm). The coordinates of all atoms were written to file every 100 ps over a simulation length of 0.1 µs.

To generate starting structures for the production run, we calculated the RMSD of all main chain atoms and all carbon atoms closest to the main chain (within one covalent bond) to the first frame of the simulation (after least-square fit on the backbone) for each prerun using 'gmx rms'. The resulting RMSD distribution was split into 20 equally sized intervals. Out of each interval one starting structure was taken randomly.

These starting structures containing the respective peptide as well as the solvent, were then equilibrated under *NVT* and *NpT* conditions at temperature T=300 K for 200 ps and 300 ps, respectively. In both equilibration steps, periodic boundary conditions were applied in all directions and position restraints were used on the peptide.

Subsequently, the systems were simulated under the same conditions as described for the pre-runs except for the following changes: The temperature was set to T = 300 K. Only the solute's coordinates were written to file every 1 ps. For each structure taken from the prerun, one trajectory with a simulation length of 1 µs was produced, yielding in 20 µs of total simulation time per peptide (see Supplementary Table 5).

3.2.2 Analyses

The analyses performed for the peptides **4a**, **4b**, **3b** and **3c** are described below. To make the visual analysis of the trajectories easier, we centered the peptides in the box in each frame (GROMACS 2019.4 'gmx trjconv' with option '-pbc mol -center') and applied a translational and rotational fit, which included a least-square fit on the C_α atoms ('-fit rot+trans').

3.2.2.1 Plane angle analysis

The structures of the peptides can be described as a macrolactam that consists of two half cyclic subunits, cycle A and B, with cycle A being Cys^8-Asn^1-Hyp^2-Ile^3-Trp^4 and cycle B being Trp^4-Gly^5-Ile^6-Gly^7-Cys^8. Both subunits share Trp^4 and Cys^8 as common amino acids and they are additionally linked via the tryptathionine bridge, which consists of the side chains of Trp^4 and Cys^8 (see Supplementary Figures 3, 12, 20).

For the evaluation of the positioning of the tryptathionine bridge in relation to the macrolactam, a simplified model was created. Based on the C_α atoms of Trp^4 and Cys^8 as well as reference points out of each subunit, three planes were defined each of which represents one subunit of the peptide:

Plane E_A representing cycle A, is defined on C_α (Trp^4), C_α (Cys^8) and C_α (Hyp^2).

Plane E_B representing cycle B, is defined on C_α (Trp^4), C_α (Cys^8) and C_α (Ile^6).

Plane E_C representing the tryptathionine bridge, is defined on C_α (Trp^4), C_α (Cys^8) and C_δ (Trp^4), where C_δ (Trp^4) is the carbon atom directly linked to the sulfur atom of Cys^8.

The positioning of the tryptathione bridge was judged based on the angles between the planes E_A, E_B and E_C in **clockwise** rotation (see Figure 2c and Supplementary Figures 3, 20). In the following, the mathematical proceeding is described:

First, the position $\mathbf{r}_0 = (x_0, y_0, z_0)^T$ of C_α (Cys^8) was defined as reference point for all three planes. All other atom positions $\mathbf{r}_{0,i} = (x_{0,i}, y_{0,i}, z_{0,i})^T$ were described in relation to $\mathbf{r}_0(C_{\alpha,Cys8})$:

$$\mathbf{r}'_i = \mathbf{r}_{0,i} - \mathbf{r}_0(C_{\alpha,Cys8}). \qquad (1)$$

The resulting positions $\mathbf{r}'_i$ were used to define the normal vectors of the planes E_A, E_B and E_C:

$$E_A: \quad \mathbf{n}_A = \mathbf{r}'_i(C_{\alpha,Trp4}) \times \mathbf{r}'_i(C_{\alpha,Hyp2}),$$

$$E_B: \quad \mathbf{n}_B = \mathbf{r}'_i(C_{\alpha,Trp4}) \times \mathbf{r}'_i(C_{\alpha,Ile6}), \qquad (2)$$

$$E_C: \quad \mathbf{n}_C = \mathbf{r}'_i(C_{\alpha,Trp4}) \times \mathbf{r}'_i(C_{\delta,Trp4}).$$

The scalar product of two normal vectors $\mathbf{n}_k$ and $\mathbf{n}_l$ yields the cosine of the angle between the planes E_k and E_l:

$$\cos(\theta_{k,l}) = (\mathbf{n}_k \cdot \mathbf{n}_l) / (|\mathbf{n}_k| \cdot |\mathbf{n}_l|) = {}^{x}/_{y} \qquad (3)$$

out of which $\theta_{k,l}$ can be calculated using the arccos(x) function and defining limiting cases:

$$\theta_{k,l} = \arccos({}^{x}/_{y}) \quad \text{if } 0 \le \theta_{k,l} < \pi,$$

$$\theta_{k,l} = 2\pi - \arccos({}^{x}/_{y}) \quad \text{if } \pi \le \theta_{k,l} < 2\pi. \qquad (4)$$

with ${}^{x}/_{y}$ as defined in eq. (2). k and l represent the circular subunits A, B or C.

All atom positions were extracted from the trajectories using the feature 'mdtraj.load'of the python library mdtraj 1.9.3.[15] The normal vectors and angles between the planes were computed using the functions linalg.norm(), dot(), cross() and arccos().[18]

3.2.2.2 Distance calculations

For two atoms i and j, the Euclidean distance was calculated as follows:

$$\mathbf{d}_{i,j} = | \mathbf{r}_j - \mathbf{r}_i | = [(x_j - x_i)^2 + (y_j - y_i)^2 + (z_j - z_i)^2]^{1/2} \quad (5)$$

with $\mathbf{r}_i = (x_i, y_i, z_i)^T$ as the atom position of atom i extracted from the MD trajectories using the feature 'mdtraj.load' of the python library mdtraj 1.9.3. [7]

3.2.2.3 Hydrogen bonds

Population analyses for single hydrogen bonds

Hydrogen bonds were analyzed with GROMACS 2019.4[10a] simulation package using 'gmx hbond'. After assigning each atom to either the side or the main chain of its residue with ‚gmx make_ndx', all interactions were classified as side ('s) or main ('m') chain interactions of the involved residues. The hydrogen bonds were evaluated based on their relative occurrence over the total simulation length. Hydrogen bonds with a relative occurrence greater than 50% in at least one simulation were added to a list 'hbond_best'. For the hydrogen bonds of this list, the population average over all replicas of the respective peptide was calculated. Hydrogen bonds of 'hbond_best' with a population of at least 10% were considered significant.

Population analyses for hydrogen bond combinations

The time series of the hydrogen bonds only contain '0' and '1' as values: '1' if the respective hydrogen bond is present, '0' if not. Based on the time series of the individual hydrogen bonds, the populations of all possible combinations of them can be achieved by:

$$X(t) = \Sigma^{N}_{k}\ 2^k\ t_k(i) \quad (6)$$

where i denotes the i-th time step of an MD trajectory X that is assigned to the states $N_s = \Sigma_k 2^k$ with N as the number of different hydrogen bonds. $t_k(i)$ denotes the time series of hydrogen bond k at time step i.

For **4a**, the time series of the following hydrogen bonds were considered:

A : Asn^1(m) – Gly^5(m),

B : Ile^3(m) – Asn^1(s),

C : Trp^4(m) – Asn^1(m),

D : Gly^5(m) – Asn^1(m),

E : Cys^8(m) – Gly^5(m),

yielding 31 different combinations according to eq. (6):

H-Bonds	A	B	C	D	E
N_S	k_0	k_1	k_2	k_3	k_4
0	0	0	0	0	0
1	1	0	0	0	0
2	0	1	0	0	0
3	1	1	0	0	0
4	0	0	1	0	0
5	1	0	1	0	0
6	0	1	1	0	0
7	1	1	1	0	0
8	0	0	0	1	0
9	1	0	0	1	0
10	0	1	0	1	0
11	1	1	0	1	0
12	0	0	1	1	0
13	1	0	1	1	0
14	0	1	1	1	0
15	1	1	1	1	0
16	0	0	0	0	1
17	1	0	0	0	1
18	0	1	0	0	1
19	1	1	0	0	1
20	0	0	1	0	1
21	1	0	1	0	1
22	0	1	1	0	1
23	1	1	1	0	1
24	0	0	0	1	1
25	1	0	0	1	1
26	0	1	0	1	1
27	1	1	0	1	1
28	0	0	1	1	1
29	1	0	1	1	1
30	0	1	1	1	1
31	1	1	1	1	1

For **4b**, the time series of the following hydrogen bonds were considered:

A : ‘Ile^{6}(m)-Ile^{3}(m)’

B : ‘Gly^{7}(m)-Ile^{3}(m)’

C : ‘Cys^{8}(m)-Ile^{3}(m)’

According to eq. (6) (*N* = 3), there are 7 different combinations for A, B and C:

H-Bonds	A	B	C
N_S	k_0	k_1	k_2
0	0	0	0
1	1	0	0
2	0	1	0
3	1	1	0
4	0	0	1
5	1	0	1
6	0	1	1
7	1	1	1

For **4a** and **4b**, the populations of the hydrogen bond combinations were then analyzed with reference to the trajectory length (1 µs per trajectory).

3.2.2.4 Linear correlations between time series of observables

For the investigation of the linear correlation between two variables, the Pearson correlation coefficients were calculated using the function corrcoef() of the numpy library.[22]

3.2.2.5 RMSD / RMSF calculations

RMSD and RMSF calculations were done with GROMACS 2019.4 [12a] simulation package using 'gmx rms' and 'gmx rmsf'. Below, the exact input and reference structure selections are explained for the different peptides:

4a and **4b**

The trajectories were fitted (translation and rotation) to the respective crystal structure using 'gmx trjconv -fit rot+trans'. Afterwards, the RMSF was calculated on all atoms towards the crystal structures. Please note, for this step, the crystal structure file and the structure file of the simulations had to be brought into line with each other.

The RMSD was calculated i) on all atoms and ii) on all main chain and carbon atoms closest to the main chain (within one covalent bond) were calculated. For both RMSD calculations, the least-squares fit was applied on the backbone atoms.

3b and **3c**

The trajectories were fitted ('gmx trjconv -fit rot+trans') to the starting structure of the respective simulation focusing on the cycle B (see Figure 2b and Supplementary Figures 3, 20), i.e. the cycle of the molecule that was already formed. As a result, all frames of a trajectory show the same orientation of that cycle in space. The RMSF was calculated on all atoms towards the highest-probability structure of the MD ensemble

(see 3.2.2.9). The RMSD was calculated on i) all atoms and ii) on the existing ring only. In both cases, the least-squares fit was applied on the existing ring.

Indices

To compare the RMSF of the compounds **4a**, **4b**, **3b** and **3c**, the atoms of the structures were re-organized such that the order of the first 114 atoms (excluding caps) was identical in all structure files. The resulting structure files are published on Zenodo (DOI: 10.5281/zenodo.6974777) [23].

3.2.2.6 Backbone and dihedral angles

Time series of dihedral angles were extracted from the simulation data with GROMACS 2019.4 [10a] simulation package using 'gmx gangle'. The backbone angles backbone angles (φ,ψ) of a residue *i* were defined as follows:

φ: $\angle$ (C_{i-1}, N_i, $C_{\alpha,i}$, C_i)

ψ: $\angle$ (N_i, $C_{\alpha,i}$, C_i, N_{i+1})

For non-glycine residues, the dihedral angles of the side chain towards the main chain were defined as:

χ: $\angle$ (N_i, $C_{\alpha,i}$, $C_{\beta,i}$, $C_{\gamma,i}$)

3.2.2.7 Solvent accessible surface area

The solvent accessible surface area of **4b** was calculated with 'md.shrake_rupley' of the python library mdtraj 1.9.3.[7] ‚md.shrake_rupley' is a new implementation of an algorithm from Shrake and Rupley from 1973.[24]

As input, the atom indices of the following amide functions (N,H) were considered:

(Residue: N, H)

Asn1: 1, 10

Ile3: 30, 39

Trp4: 49, 68

Gly5: 72, 77

Ile6: 79, 88

Gly7: 98, 103

Cys8: 105, 112

3.2.2.8 Ground state energies

All quantum-chemical calculations were performed using ORCA version 5.0.0[25]. Using Grimme's GFN2-xTB method[26] interfaced with ORCA, single-point energies were calculated for all frames of the MD simulation trajectories of **4a** and **4b**, respectively. The 100 structures with lowest energy were then optimized at the B3LYP-D4(BJ)/def2-TZVP[27] level of density functional theory adding implicit solvation in

water using CPCM[28] (ε=78.4) and using the RIJCOSX approximation[29] ('RIJCOSX def2/J') to accelerate the SCF calculations. For the resulting optimized structures, full TDDFT calculations were performed using the setup of the optimization ('B3LYP DEF2-TZVP D4 CPCM(WATER); 'RIJCOS def2/J'). 25 states were calculated.

3.2.2.9 Extraction of highest-probability structures

Using the hydrogen bond time series described in 3.2.2.3, structures were extracted based on the most-populated, positively correlated hydrogen bonds (Supplementary Figure 2). For **3b**, these hydrogen bonds were (population on average over 20 μs is given in brackets): 'Asn^1(m)-Trp^4(m)' (60%), 'Trp^4(m)-Asn^1(m)' (37%), 'Gly^7(m)-Trp^4(m)' (51%) and 'Cys^8(m)–Trp^4(m)' (65%). For **3c**, these hydrogen bonds were 'Trp^4(m)-Ile^6(m)' (43%), 'Gly^5(m)-Ile^6(m)' (35%) and 'Cys^8(m)-Hyp^2(m)' (43%).

Hence, for **3b** and **3c**, all frames were extracted, in which the respective hydrogen bonds were all present. The resulting sub-data sets comprised 28% (**3b**) and 31% (**3c**) of the respective entire data sets. Both sub-data sets were checked based on the RMSD (see 3.2.2.5) of the backbone from Trp^4 to Asn^1 with a preceding least-squares fit on the existing ring, only. The average RMSD of the sub-data sets amounted to 0.05 nm (**3b**) and 0.03 nm (**3c**). Consequently, the sub-data sets were considered unified and representative of the highest-probability structures of **3b** and **3c**.

The highest probability structures are publicly available on Zenodo (DOI: 10.5281/zenodo.6974777) [23].

3.2.2.10 Determining transition times (solely for **4a**):

The time for the transition between the starting structure and the crystal structure was estimated as follows: The trajectories were discretized into three states according to 3.2.2.3: state '1' comprises the hydrogen bond set that is in agreement with the crystal structure ('Asn^1(m)-Gly^5(m)', 'Ile^3(m)-Asn^1(s)', 'Trp^4(m)-Asn^1(m)', 'Gly^5(m)-Asn^1(m)', 'Cys^8(m)-Gly^5(m)'). State '2' comprises hydrogen bonds that are mutually exclusive to hydrogen bond set '1' ('Asn^1(m)-Hyp^2(m)', 'Cys^8(m)-Hyp^2(m)'). All frames that could not be unambiguously assigned to '1' or '2' were considered as noise ('0', Fig. S11a). These frames denote short-term transitions out of the two conformations. The transition time is the moment when the system reaches state '1' if the system was in '2' before and hasn't visited '1' by that time. Based on the discretized trajectories, the transition times were calculated using python (pseudo-code):

```
> transition = []
># Loop over all replicas:
>for rep in range(20):
>    time = np.arange(0,len(traj),1)
```

```
>   here_set1 = np.where(traj==1)[0]
>   here_set2 = np.where(traj==2)[0]
# If both states exist:
>   if ((np.size(here_set1)>0) and (np.size(here_set2)>0)):
            m1 = np.min(here_set1)
            m2 = np.max(here_set2)
            if m1 < m2:
                    break
            else:
                    transition.append(m1)
>   else:
            transition.append([ ])
```

3.2.2.11 Comparison between NMR and MD based on NOE distances

Interatomic distances between protons *a* and *b* were calculated with 'gmx distance -oall' using GROMACS 2020.6 simulation package [12,30] and a list of atom indices from NMR experiments.

Considering the time series of the inter-proton distance $r_{a,b}(t)$, the following averages were calculated for each replica:[31,32]

$$R^{MD}_{a,b} = \langle z(t) \rangle^{1/6} = [\ (^1/_N)\ \Sigma^N_{f=1}\ z_f\ ]^{1/6} \qquad (7)$$

$$z(t) = (\ r_{a,b}(t)\ )^{-6} \qquad (8)$$

with N=10^6 as the number of structures *f* in each replica. The deviations from experimental inter-proton distances (NMR NOE and crystal structure) were then determined by:

$$\Delta(R_{a,b}) = R^{MD}_{a,b} - R^{NMR}_{a,b} \qquad (9)$$

calling $\Delta(R_{a,b})$ 'NOE distance violation'. Any distance with $\Delta(R_{a,b})$ > 0.1 nm was considered 'violated', i.e. the MD trajectory deviates from the NMR ensemble in this distance beyond the experimental uncertainty.

The distribution of these distance violations over all replicas (n=20, 20 µs simulation time in total) was visualized as box-and-whisker plots using 'pyplot.boxplot' from the python library 'matplotlib' (version 3.5.3).

3.3 NMR spectra

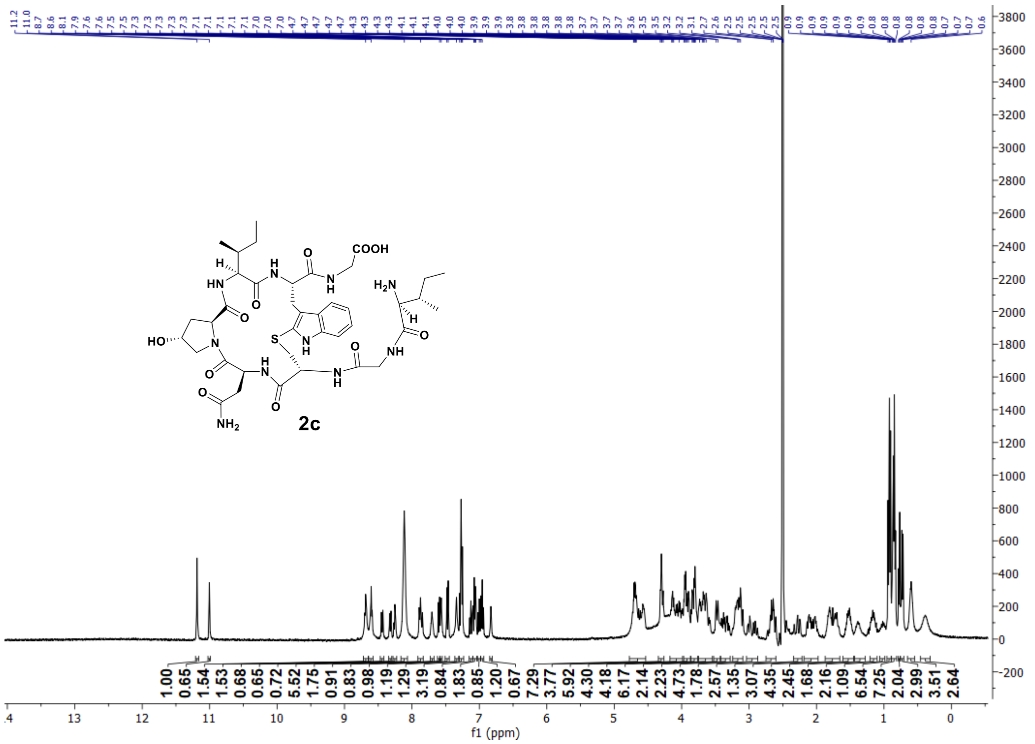

Supplementary Figure 23. ^{1}H NMR Spectrum of peptide **2c**

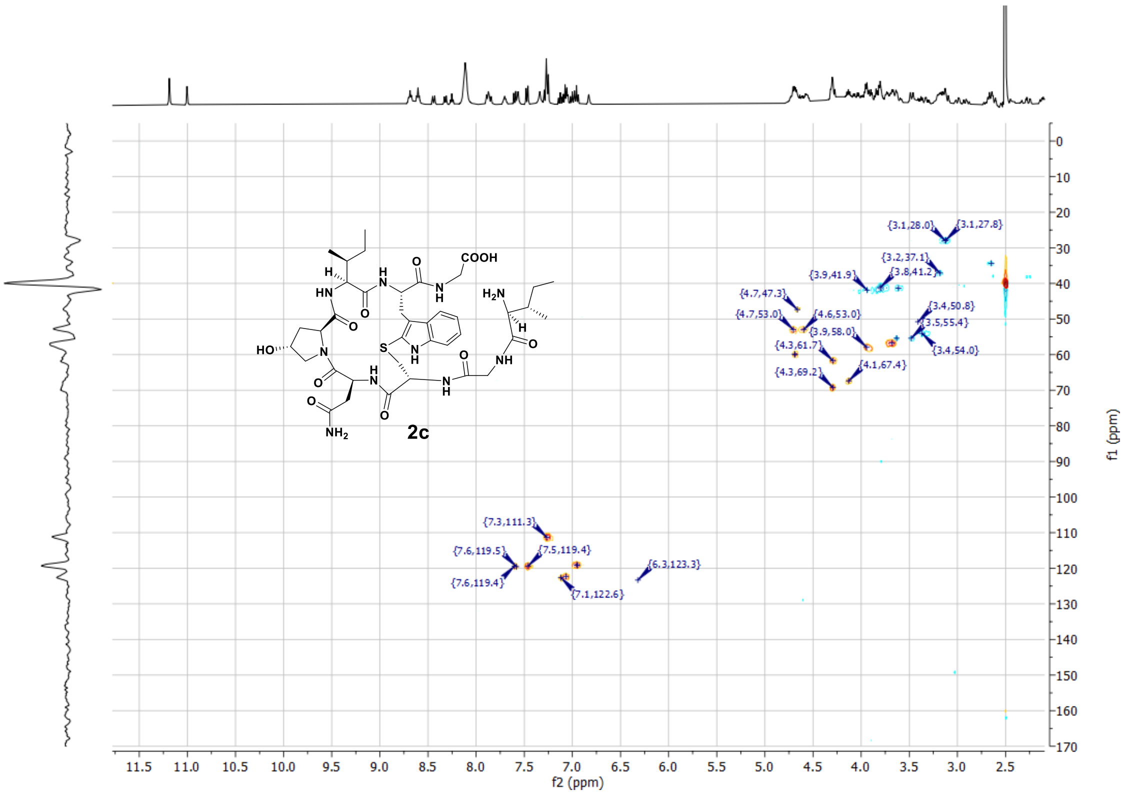

Supplementary Figure 24. HSQC-ed Spectrum of peptide **2c**

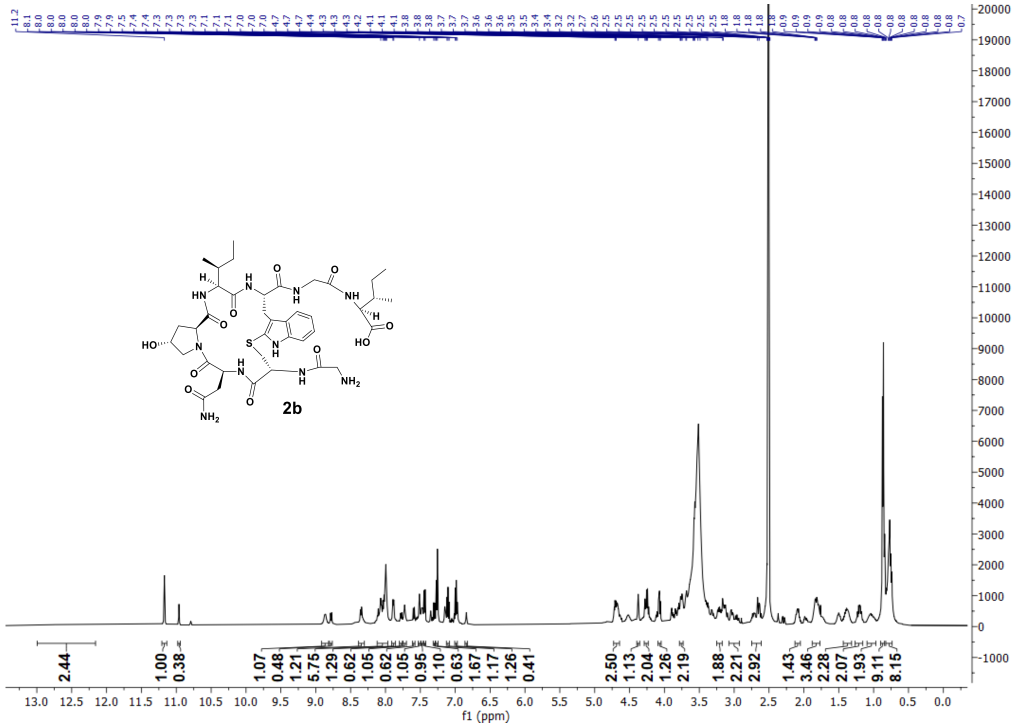

Supplementary Figure 25. ^{1}H NMR Spectrum of peptide **2b**

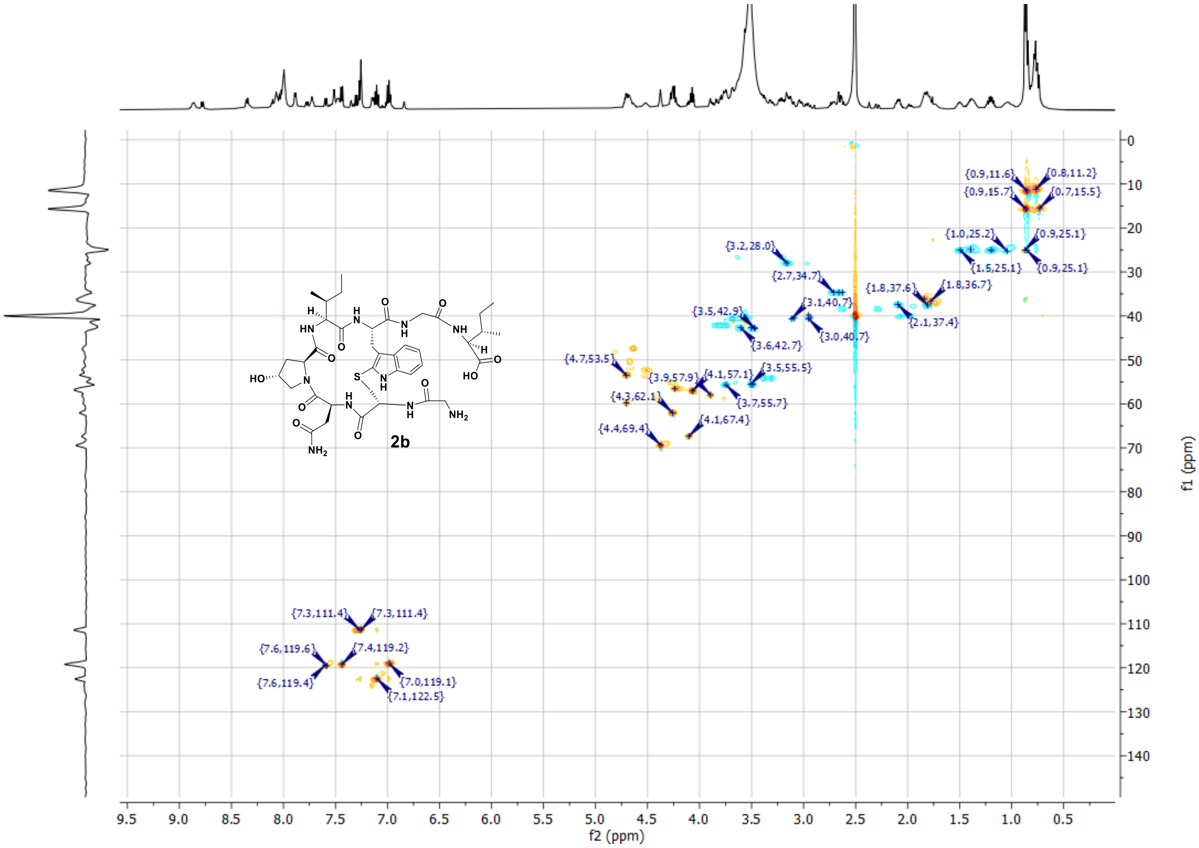

Supplementary Figure 26. HSQC-ed Spectrum of peptide **2b**

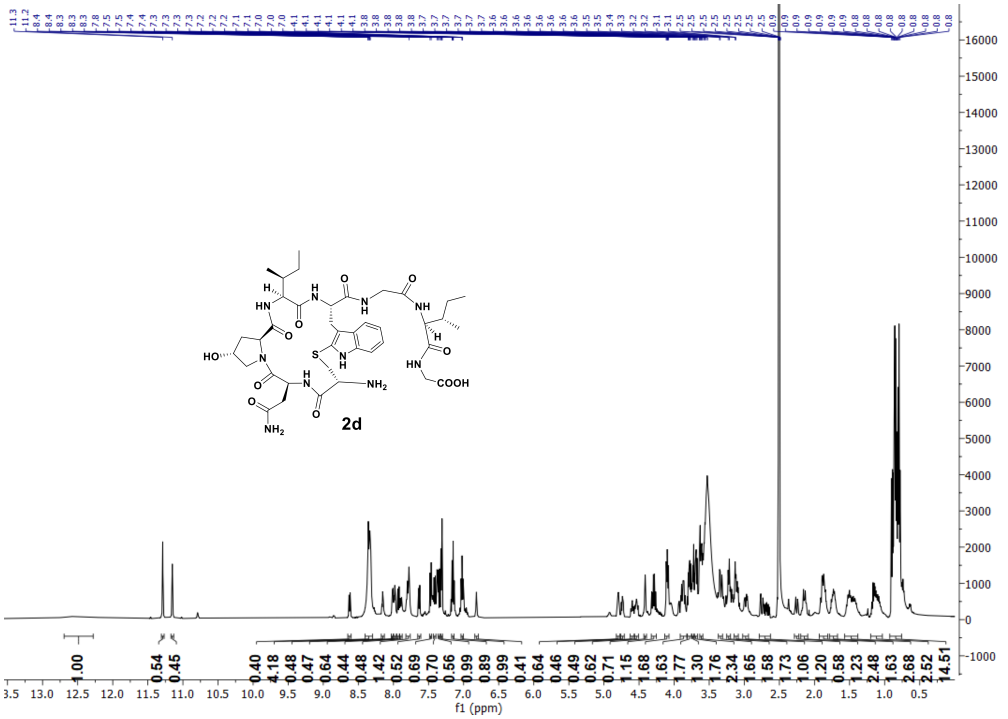

Supplementary Figure 27. ^{1}H NMR Spectrum of peptide **2d**

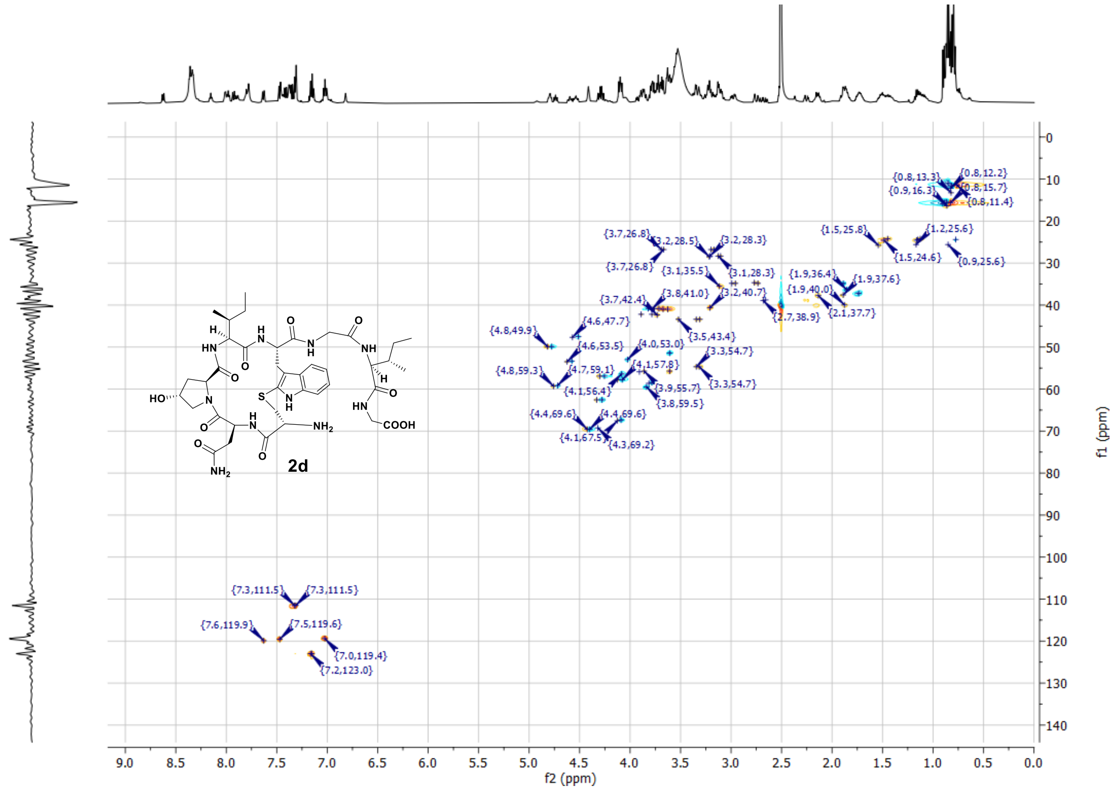

Supplementary Figure 28. HSQC-ed Spectrum of peptide **2d**

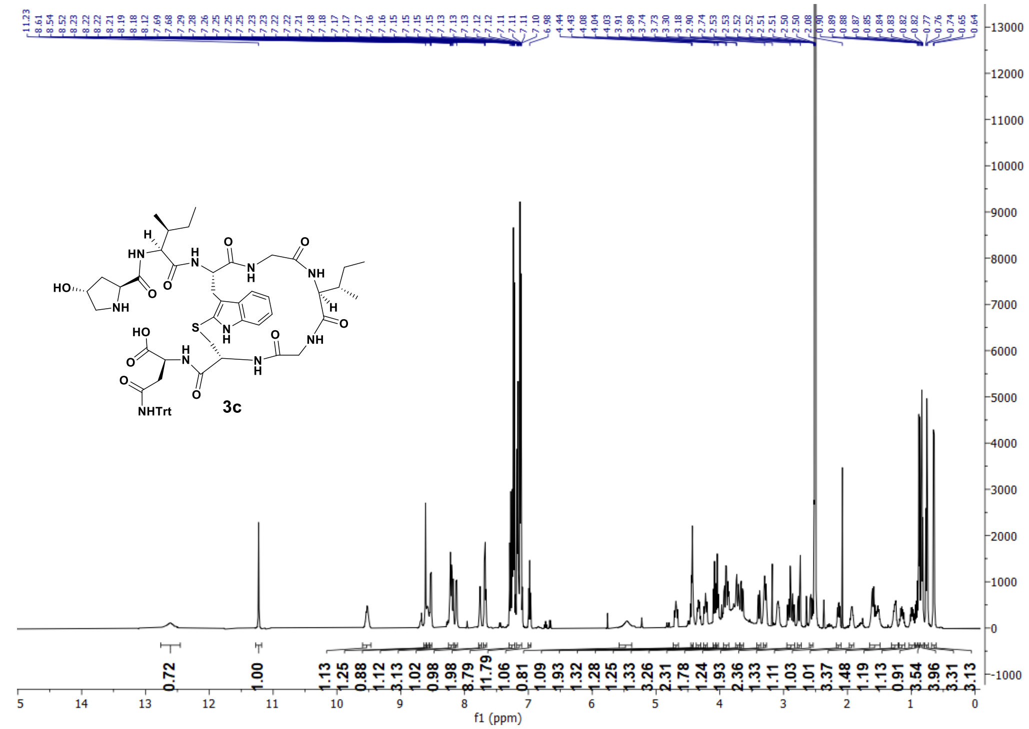

Supplementary Figure 29. ^{1}H NMR Spectrum of peptide **3c**

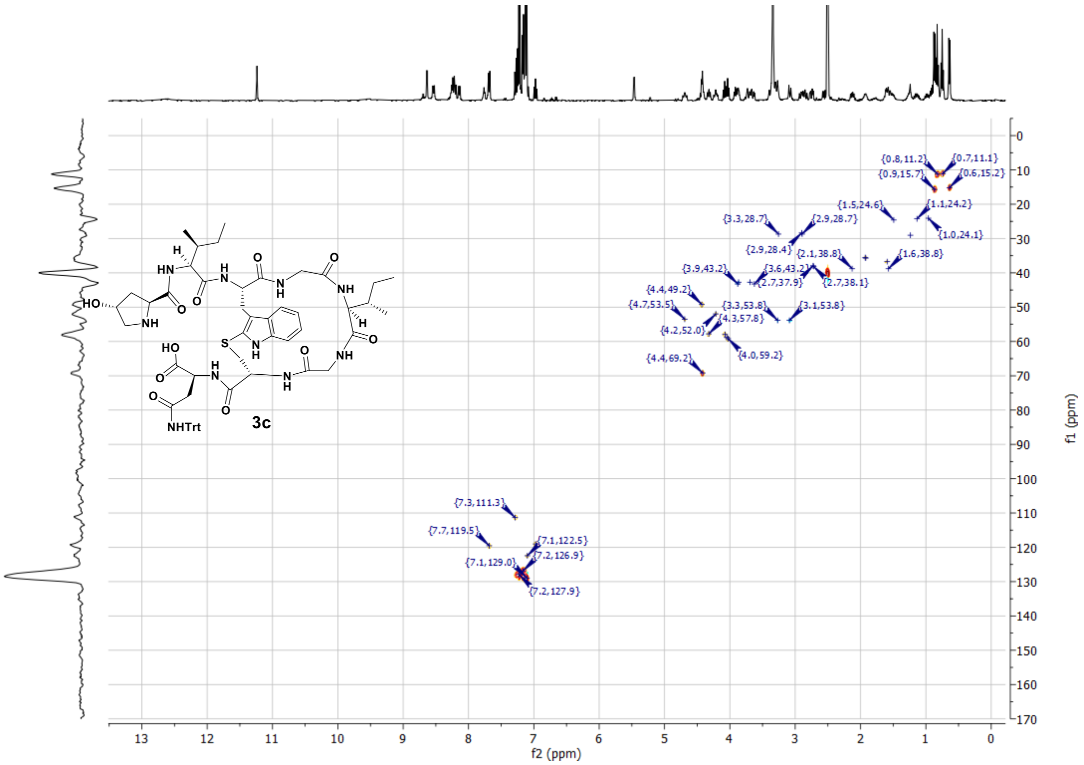

Supplementary Figure 30. HSQC-ed Spectrum of peptide **3c**

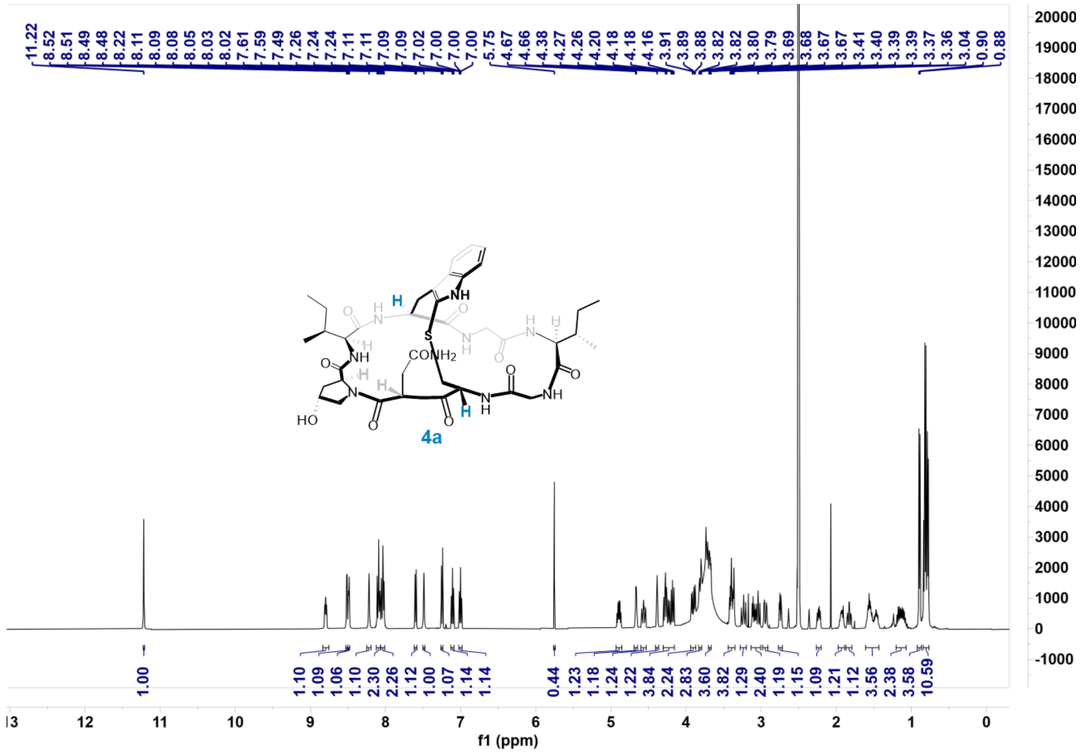

Supplementary Figure 31. ^{1}H NMR Spectrum of peptide **4a**

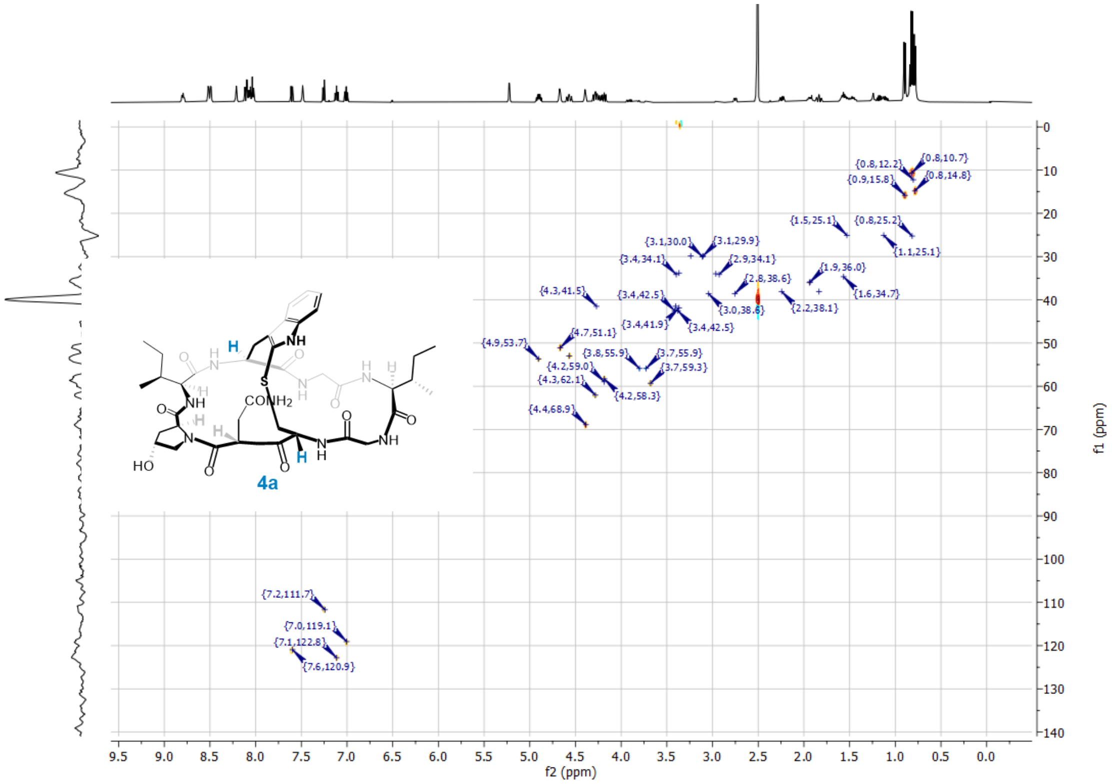

Supplementary Figure 32. HSQC-ed Spectrum of peptide **4a**

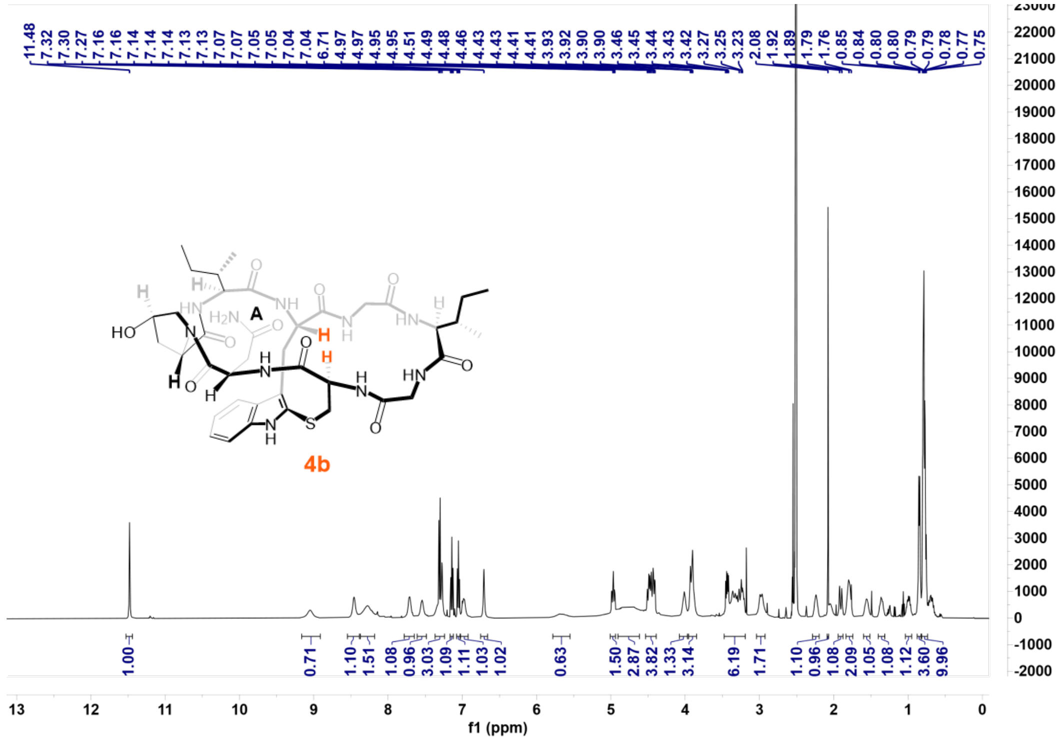

Supplementary Figure 33. ^{1}H NMR Spectrum of peptide **4b**

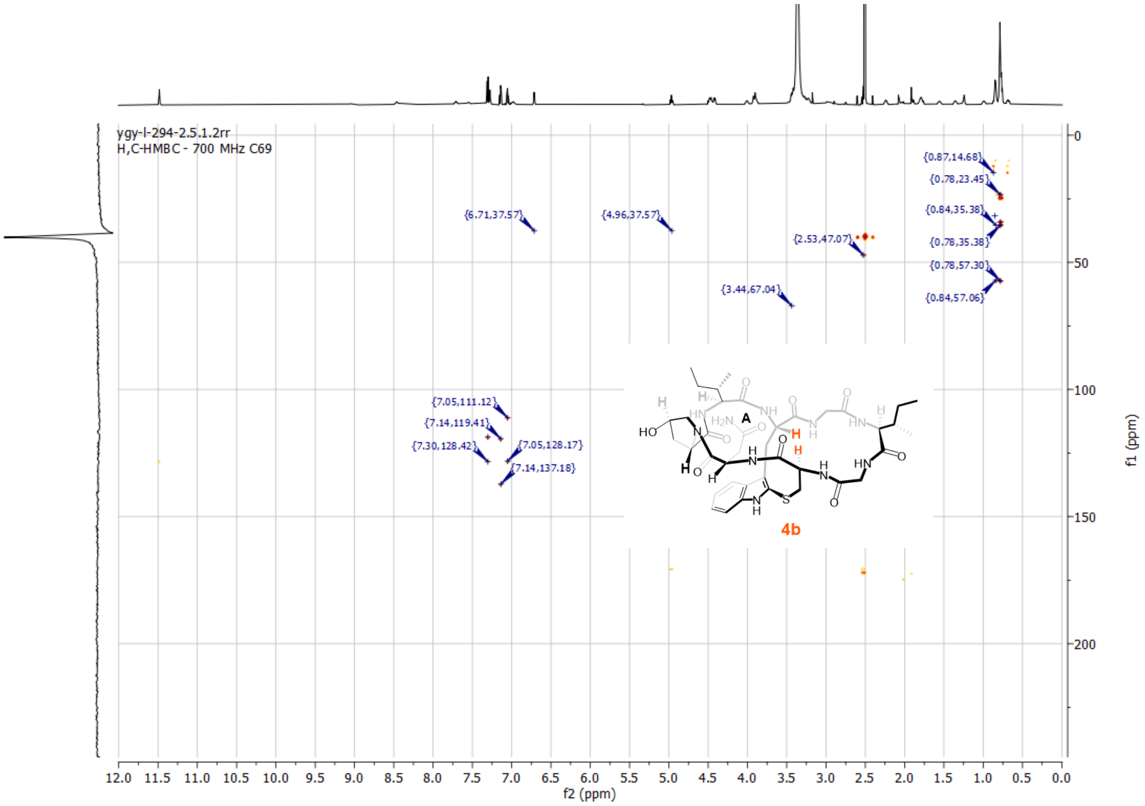

Supplementary Figure 34. HSQC-ed Spectrum of peptide **4b**

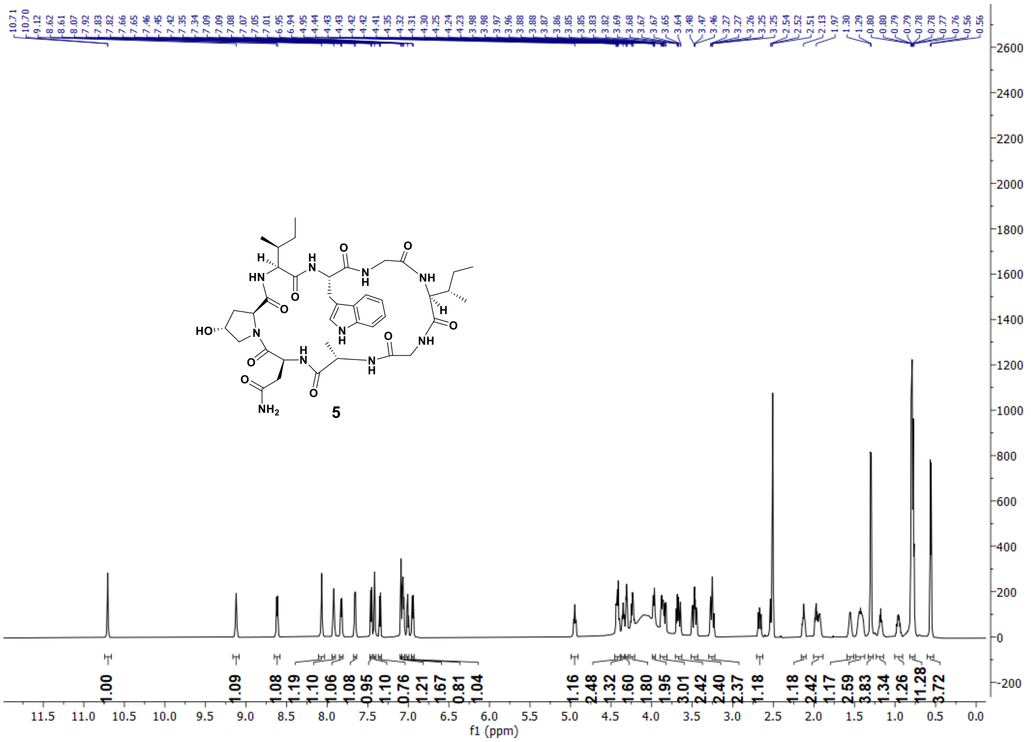

Supplementary Figure 35. ^{1}H NMR Spectrum of peptide **5**

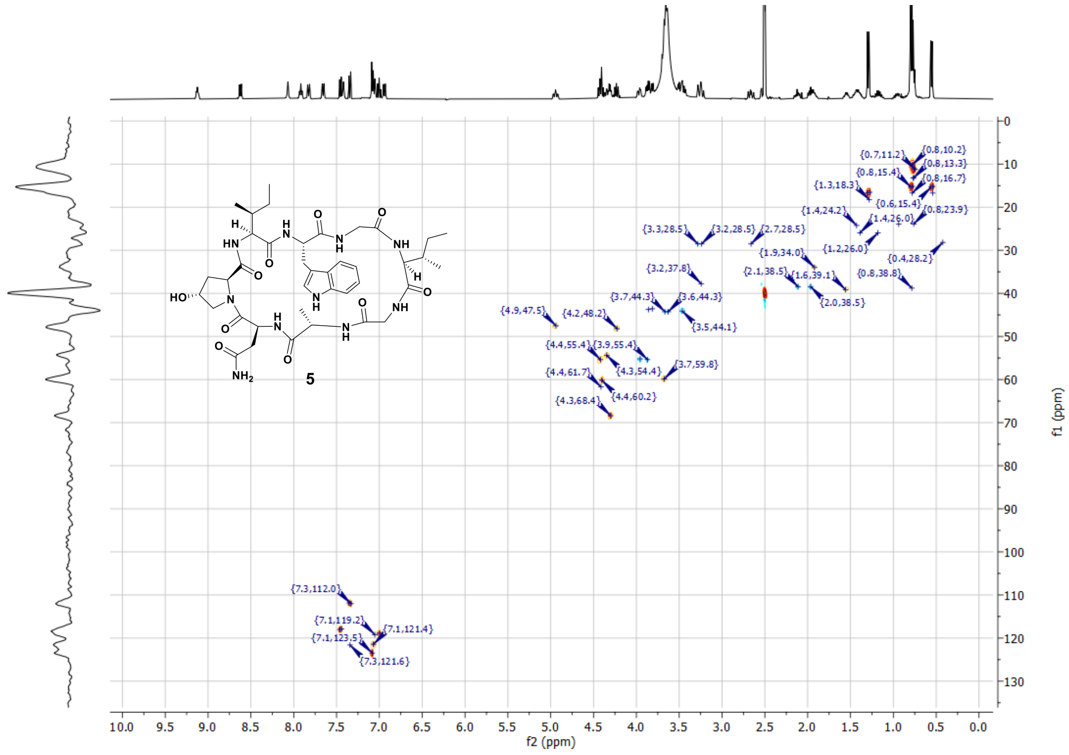

Supplementary Figure 36. HSQC-ed Spectrum of peptide **5**

3.4 X-Ray

Supplementary Table 6: Crystal data and structure refinement for cu-4372.

Identification code	cu-4372	
Empirical formula	C39 H62 N10 O14 S	
Formula weight	927.05	
Temperature	150.01(10) K	
Wavelength	1.54184 Å	
Crystal system	Trigonal	
Space group	R3 (No.146)	
Unit cell dimensions	a = 32.9063(5) Å	a= 90°.
	b = 32.9063(5) Å	b= 90°.
	c = 11.7975(2) Å	g = 120°.
Volume	11063.1(3) $Å^3$	
Z	9	
Density (calculated)	1.252 Mg/m^3	
Absorption coefficient	1.180 mm^{-1}	
F(000)	4446	
Crystal size	0.21 x 0.13 x 0.12 mm^3	
Theta range for data collection	2.69 to 67.44°.	
Index ranges	-39<=h<=38, -38<=k<=39, -14<=l<=13	
Reflections collected	14947	
Independent reflections	6962 [R(int) = 0.0270]	
Completeness to theta = 67.44°	99.9 %	
Absorption correction	Semi-empirical from equivalents	
Max. and min. transmission	0.8714 and 0.7897	

Refinement method	Full-matrix least-squares on F^2
Data / restraints / parameters	6962 / 39 / 621
Goodness-of-fit on F^2	1.054
Final R indices [I>2sigma(I)]	R1 = 0.0399, wR2 = 0.1042
R indices (all data)	R1 = 0.0444, wR2 = 0.1087
Absolute structure parameter	0.041(18)
Largest diff. peak and hole	0.814 and -0.237 e.$Å^{-3}$

Supplementary Table 7: Atomic coordinates (x 10^4) and equivalent isotropic displacement parameters ($Å^2$x 10^3) for cu-4372. U(eq) is defined as one third of the trace of the orthogonalized U^{ij} tensor.

	x	y	z	U(eq)
S(1)	4706(1)	5796(1)	11649(1)	34(1)
O(2)	6043(1)	6329(1)	5008(2)	29(1)
O(3)	5131(1)	5463(1)	6181(2)	39(1)
O(4)	6284(1)	7981(1)	4768(2)	33(1)
O(5)	4288(1)	5842(1)	7781(2)	34(1)
O(6)	5258(1)	5188(1)	9234(2)	33(1)
O(7)	5996(1)	6306(1)	9408(2)	31(1)
O(8)	2594(1)	4132(1)	8032(2)	42(1)
O(10)	5384(1)	7093(1)	8389(2)	37(1)
N(12)	4798(1)	5505(1)	9005(2)	26(1)
N(13)	4096(1)	4678(1)	8003(2)	33(1)
O(14)	3216(1)	5824(1)	8948(2)	48(1)
N(15)	5922(1)	6849(1)	5975(2)	26(1)
O(16)	4101(1)	4404(1)	6260(2)	47(1)
N(18)	4221(1)	6358(1)	8911(2)	34(1)
N(19)	5578(1)	6146(1)	7772(2)	25(1)
N(20)	3328(1)	5218(1)	9434(2)	35(1)
N(21)	5219(1)	6741(1)	11963(2)	33(1)
C(22)	5460(1)	5384(1)	6285(2)	30(1)
C(23)	5425(1)	6951(1)	7456(2)	26(1)

C(24)	5916(1)	7098(1)	7000(2)	25(1)
C(25)	4886(1)	5152(1)	8893(2)	28(1)
N(26)	3323(1)	4733(1)	7594(2)	33(1)
C(27)	5973(1)	6466(1)	5918(2)	25(1)
N(29)	5057(1)	6702(1)	6770(2)	32(1)
C(30)	5619(1)	6144(1)	8911(2)	23(1)
N(31)	5422(1)	4976(1)	6011(2)	41(1)
C(32)	5984(1)	7561(1)	5375(3)	28(1)
C(33)	5926(1)	5742(1)	6788(2)	28(1)
C(34)	5149(1)	5946(1)	9535(2)	24(1)
C(35)	5962(1)	6212(1)	7031(2)	26(1)
C(36)	4530(1)	4703(1)	8362(3)	38(1)
C(37)	3460(1)	5676(1)	9347(3)	34(1)
C(38)	4483(1)	6514(1)	11365(3)	32(1)
C(39)	4593(1)	7351(1)	11559(3)	36(1)
C(40)	5513(1)	7601(1)	12210(3)	41(1)
C(41)	3434(1)	4484(1)	6740(3)	36(1)
C(43)	3908(1)	4523(1)	6977(3)	35(1)
C(44)	5186(1)	7140(1)	11938(2)	32(1)
C(45)	6146(1)	7609(1)	6598(2)	28(1)
C(46)	6001(1)	7131(1)	4950(2)	28(1)
C(47)	3399(1)	4631(1)	5523(3)	43(1)
C(48)	4799(1)	6367(1)	11620(3)	32(1)
C(49)	3990(1)	6226(1)	10934(3)	36(1)
C(50)	2874(1)	4839(1)	9070(3)	36(1)

C(52)	5371(1)	7930(1)	12164(3)	43(1)
C(53)	4362(1)	6244(1)	7961(3)	30(1)
C(54)	4725(1)	7011(1)	11586(2)	31(1)
C(55)	5205(1)	5884(1)	10802(2)	30(1)
C(56)	4592(1)	6627(1)	7070(3)	34(1)
C(57)	3969(1)	6003(1)	9765(3)	32(1)
C(58)	3736(1)	5149(1)	5324(3)	47(1)
C(59)	2919(1)	4536(1)	8182(3)	32(1)
C(60)	2890(1)	4491(2)	5275(4)	67(1)
C(61)	4914(1)	7809(1)	11856(3)	42(1)
C(63)	3778(2)	5296(2)	4088(4)	66(1)
O(1)	6755(1)	6101(1)	9330(2)	36(1)
O(9)	5292(1)	9041(1)	10246(2)	44(1)
O(11)	4964(1)	6076(1)	14786(2)	51(1)
C(7)	3787(1)	6380(1)	6355(4)	62(1)
O(15)	4394(1)	4685(1)	11754(3)	58(1)
C(6)	4280(1)	6499(2)	6027(3)	49(1)
C(1)	4472(2)	6945(2)	5298(5)	47(1)
C(2)	4206(2)	6839(2)	4183(5)	56(1)
C(1X)	4525(4)	6756(4)	4800(9)	45(2)
C(2X)	4642(4)	7259(4)	4975(10)	50(2)

Supplementary Table 8: Bond lengths [Å] and angles [°] for cu-4372.

S(1)-C(48)	1.746(3)
S(1)-C(55)	1.816(3)
O(2)-C(27)	1.229(3)
O(3)-C(22)	1.240(4)
O(4)-C(32)	1.426(3)
O(5)-C(53)	1.238(4)
O(6)-C(25)	1.238(3)
O(7)-C(30)	1.227(3)
O(8)-C(59)	1.233(4)
O(10)-C(23)	1.229(4)
N(12)-C(25)	1.337(4)
N(12)-C(34)	1.468(4)
N(13)-C(43)	1.338(4)
N(13)-C(36)	1.454(4)
O(14)-C(37)	1.220(4)
N(15)-C(27)	1.355(4)
N(15)-C(24)	1.466(3)
N(15)-C(46)	1.467(4)
O(16)-C(43)	1.232(4)
N(18)-C(53)	1.336(4)
N(18)-C(57)	1.449(4)
N(19)-C(30)	1.351(4)
N(19)-C(35)	1.462(3)

N(20)-C(37)	1.346(4)
N(20)-C(50)	1.453(4)
N(21)-C(44)	1.369(4)
N(21)-C(48)	1.372(4)
C(22)-N(31)	1.325(4)
C(22)-C(33)	1.513(4)
C(23)-N(29)	1.342(4)
C(23)-C(24)	1.534(4)
C(24)-C(45)	1.535(4)
C(25)-C(36)	1.488(4)
N(26)-C(59)	1.344(4)
N(26)-C(41)	1.455(4)
C(27)-C(35)	1.547(4)
N(29)-C(56)	1.468(4)
C(30)-C(34)	1.533(4)
C(32)-C(45)	1.519(4)
C(32)-C(46)	1.526(4)
C(33)-C(35)	1.516(4)
C(34)-C(55)	1.531(4)
C(37)-C(57)	1.551(4)
C(38)-C(48)	1.380(4)
C(38)-C(54)	1.440(4)
C(38)-C(49)	1.501(4)
C(39)-C(61)	1.386(5)
C(39)-C(54)	1.389(4)

C(40)-C(52)	1.378(5)
C(40)-C(44)	1.390(5)
C(41)-C(43)	1.527(5)
C(41)-C(47)	1.537(5)
C(44)-C(54)	1.417(4)
C(47)-C(58)	1.516(5)
C(47)-C(60)	1.529(5)
C(49)-C(57)	1.547(4)
C(50)-C(59)	1.507(4)
C(52)-C(61)	1.399(5)
C(53)-C(56)	1.520(4)
C(56)-C(6)	1.521(5)
C(58)-C(63)	1.521(5)
C(7)-C(6)	1.518(5)
C(6)-C(1)	1.540(6)
C(6)-C(1X)	1.667(11)
C(1)-C(2)	1.521(9)
C(1X)-C(2X)	1.517(16)
C(48)-S(1)-C(55)	99.14(13)
C(25)-N(12)-C(34)	119.7(2)
C(43)-N(13)-C(36)	122.6(3)
C(27)-N(15)-C(24)	127.1(2)
C(27)-N(15)-C(46)	118.9(2)
C(24)-N(15)-C(46)	112.0(2)

C(53)-N(18)-C(57)	119.7(2)
C(30)-N(19)-C(35)	120.9(2)
C(37)-N(20)-C(50)	123.6(3)
C(44)-N(21)-C(48)	109.1(2)
O(3)-C(22)-N(31)	122.6(3)
O(3)-C(22)-C(33)	121.8(3)
N(31)-C(22)-C(33)	115.5(3)
O(10)-C(23)-N(29)	122.4(3)
O(10)-C(23)-C(24)	119.6(2)
N(29)-C(23)-C(24)	117.8(2)
N(15)-C(24)-C(23)	114.9(2)
N(15)-C(24)-C(45)	102.7(2)
C(23)-C(24)-C(45)	108.5(2)
O(6)-C(25)-N(12)	121.2(3)
O(6)-C(25)-C(36)	118.8(3)
N(12)-C(25)-C(36)	120.0(3)
C(59)-N(26)-C(41)	123.4(3)
O(2)-C(27)-N(15)	121.2(2)
O(2)-C(27)-C(35)	120.1(2)
N(15)-C(27)-C(35)	118.7(2)
C(23)-N(29)-C(56)	120.1(3)
O(7)-C(30)-N(19)	123.7(3)
O(7)-C(30)-C(34)	122.8(2)
N(19)-C(30)-C(34)	113.4(2)
O(4)-C(32)-C(45)	109.8(2)

O(4)-C(32)-C(46)	114.0(2)
C(45)-C(32)-C(46)	103.7(2)
C(22)-C(33)-C(35)	113.7(2)
N(12)-C(34)-C(55)	111.9(2)
N(12)-C(34)-C(30)	110.2(2)
C(55)-C(34)-C(30)	111.3(2)
N(19)-C(35)-C(33)	109.9(2)
N(19)-C(35)-C(27)	111.9(2)
C(33)-C(35)-C(27)	111.0(2)
N(13)-C(36)-C(25)	116.2(2)
O(14)-C(37)-N(20)	124.6(3)
O(14)-C(37)-C(57)	122.8(3)
N(20)-C(37)-C(57)	112.5(3)
C(48)-C(38)-C(54)	105.8(3)
C(48)-C(38)-C(49)	128.2(3)
C(54)-C(38)-C(49)	126.0(3)
C(61)-C(39)-C(54)	119.8(3)
C(52)-C(40)-C(44)	117.9(3)
N(26)-C(41)-C(43)	111.1(2)
N(26)-C(41)-C(47)	113.1(3)
C(43)-C(41)-C(47)	112.2(3)
O(16)-C(43)-N(13)	121.9(3)
O(16)-C(43)-C(41)	121.0(3)
N(13)-C(43)-C(41)	117.1(3)
N(21)-C(44)-C(40)	130.8(3)

N(21)-C(44)-C(54)	107.7(3)
C(40)-C(44)-C(54)	121.5(3)
C(32)-C(45)-C(24)	102.9(2)
N(15)-C(46)-C(32)	103.8(2)
C(58)-C(47)-C(60)	112.6(3)
C(58)-C(47)-C(41)	111.7(3)
C(60)-C(47)-C(41)	109.0(3)
N(21)-C(48)-C(38)	110.3(3)
N(21)-C(48)-S(1)	121.0(2)
C(38)-C(48)-S(1)	128.5(2)
C(38)-C(49)-C(57)	112.3(2)
N(20)-C(50)-C(59)	111.9(2)
C(40)-C(52)-C(61)	121.7(3)
O(5)-C(53)-N(18)	122.2(3)
O(5)-C(53)-C(56)	121.2(3)
N(18)-C(53)-C(56)	116.5(3)
C(39)-C(54)-C(44)	119.0(3)
C(39)-C(54)-C(38)	133.8(3)
C(44)-C(54)-C(38)	107.1(3)
C(34)-C(55)-S(1)	113.59(19)
N(29)-C(56)-C(53)	110.5(2)
N(29)-C(56)-C(6)	110.7(3)
C(53)-C(56)-C(6)	110.0(2)
N(18)-C(57)-C(49)	111.4(2)
N(18)-C(57)-C(37)	108.9(3)

C(49)-C(57)-C(37)	112.9(2)
C(47)-C(58)-C(63)	113.9(3)
O(8)-C(59)-N(26)	124.3(3)
O(8)-C(59)-C(50)	119.6(3)
N(26)-C(59)-C(50)	116.1(3)
C(39)-C(61)-C(52)	120.0(3)
C(7)-C(6)-C(56)	110.5(4)
C(7)-C(6)-C(1)	105.2(4)
C(56)-C(6)-C(1)	106.8(4)
C(7)-C(6)-C(1X)	122.4(5)
C(56)-C(6)-C(1X)	118.3(5)
C(1)-C(6)-C(1X)	33.6(4)
C(2)-C(1)-C(6)	110.4(5)
C(2X)-C(1X)-C(6)	103.3(9)

Symmetry transformations used to generate equivalent atoms:

Supplementary Table 9: Anisotropic displacement parameters ($Å^2$x 10^3) for cu-4372. The anisotropic displacement factor exponent takes the form: $-2p^2[h^2 a^{*2}U^{11} + ... + 2 h k a^* b^* U^{12}]$

	U^{11}	U^{22}	U^{33}	U^{23}	U^{13}	U^{12}
S(1)	33(1)	37(1)	33(1)	5(1)	11(1)	19(1)
O(2)	32(1)	30(1)	25(1)	1(1)	3(1)	16(1)
O(3)	31(1)	39(1)	42(1)	-2(1)	-4(1)	14(1)
O(4)	34(1)	28(1)	35(1)	4(1)	9(1)	13(1)
O(5)	35(1)	35(1)	38(1)	6(1)	5(1)	23(1)
O(6)	37(1)	38(1)	34(1)	-1(1)	-1(1)	26(1)
O(7)	26(1)	34(1)	31(1)	-3(1)	-2(1)	13(1)
O(8)	29(1)	32(1)	47(1)	-5(1)	6(1)	3(1)
O(10)	27(1)	43(1)	38(1)	-5(1)	8(1)	16(1)
N(12)	22(1)	23(1)	33(1)	3(1)	2(1)	12(1)
N(13)	27(1)	31(1)	42(2)	-2(1)	1(1)	14(1)
O(14)	28(1)	40(1)	66(2)	10(1)	-9(1)	11(1)
N(15)	28(1)	26(1)	25(1)	-2(1)	0(1)	14(1)
O(16)	50(1)	51(1)	45(1)	-12(1)	4(1)	29(1)
N(18)	22(1)	24(1)	50(2)	-1(1)	2(1)	6(1)
N(19)	18(1)	27(1)	28(1)	4(1)	2(1)	10(1)
N(20)	23(1)	32(1)	43(2)	-6(1)	-1(1)	10(1)
N(21)	23(1)	42(1)	36(1)	-7(1)	-6(1)	17(1)
C(22)	29(2)	28(1)	29(2)	5(1)	5(1)	12(1)
C(23)	22(1)	25(1)	33(2)	5(1)	3(1)	12(1)

C(24)	21(1)	27(1)	24(1)	-2(1)	2(1)	11(1)
C(25)	30(2)	29(1)	28(1)	3(1)	3(1)	18(1)
N(26)	24(1)	33(1)	35(1)	-2(1)	3(1)	9(1)
C(27)	18(1)	27(1)	28(1)	1(1)	3(1)	10(1)
N(29)	21(1)	39(1)	36(1)	2(1)	-2(1)	15(1)
C(30)	27(1)	19(1)	28(1)	-1(1)	-1(1)	14(1)
N(31)	42(2)	31(1)	45(2)	-9(1)	-2(1)	15(1)
C(32)	23(1)	23(1)	35(2)	2(1)	2(1)	10(1)
C(33)	24(1)	29(1)	27(1)	3(1)	3(1)	12(1)
C(34)	24(1)	24(1)	30(1)	0(1)	3(1)	16(1)
C(35)	22(1)	28(1)	25(1)	1(1)	4(1)	12(1)
C(36)	34(2)	32(2)	50(2)	0(1)	0(1)	19(1)
C(37)	24(1)	37(2)	32(2)	2(1)	4(1)	10(1)
C(38)	25(1)	36(2)	36(2)	-4(1)	-1(1)	15(1)
C(39)	34(2)	42(2)	36(2)	-7(1)	-8(1)	22(1)
C(40)	28(2)	49(2)	33(2)	-9(1)	-5(1)	10(2)
C(41)	28(2)	34(2)	36(2)	-7(1)	3(1)	8(1)
C(43)	33(2)	26(1)	37(2)	0(1)	4(1)	8(1)
C(44)	24(1)	39(2)	32(2)	-4(1)	-1(1)	16(1)
C(45)	26(1)	26(1)	30(2)	-1(1)	3(1)	9(1)
C(46)	30(2)	28(1)	24(1)	1(1)	1(1)	14(1)
C(47)	32(2)	52(2)	32(2)	-6(1)	-1(1)	13(2)
C(48)	26(1)	41(2)	32(2)	-2(1)	6(1)	19(1)
C(49)	22(1)	38(2)	44(2)	-8(1)	4(1)	11(1)
C(50)	20(1)	35(2)	45(2)	-5(1)	2(1)	6(1)

C(52)	45(2)	35(2)	36(2)	-4(1)	-2(1)	11(2)
C(53)	18(1)	34(2)	41(2)	6(1)	3(1)	15(1)
C(54)	26(1)	37(2)	31(2)	-7(1)	-2(1)	17(1)
C(55)	31(2)	34(2)	30(1)	6(1)	4(1)	21(1)
C(56)	21(1)	30(2)	52(2)	9(1)	6(1)	12(1)
C(57)	24(1)	30(2)	39(2)	-6(1)	1(1)	10(1)
C(58)	38(2)	61(2)	34(2)	5(2)	1(1)	18(2)
C(59)	26(2)	31(2)	36(2)	-2(1)	1(1)	11(1)
C(60)	34(2)	93(3)	44(2)	9(2)	-9(2)	10(2)
C(61)	50(2)	43(2)	32(2)	-5(1)	-3(1)	22(2)
C(63)	46(2)	79(3)	46(2)	12(2)	-2(2)	13(2)
O(1)	40(1)	44(1)	29(1)	-3(1)	0(1)	25(1)
O(9)	45(1)	45(1)	50(2)	-8(1)	-8(1)	28(1)
O(11)	39(1)	56(2)	53(2)	-7(1)	-14(1)	20(1)
C(7)	26(2)	44(2)	110(4)	19(2)	-11(2)	15(2)
O(15)	36(1)	68(2)	67(2)	22(1)	0(1)	23(1)
C(6)	25(2)	51(2)	62(2)	32(2)	0(2)	12(2)
C(1)	46(3)	53(3)	47(3)	21(2)	10(2)	28(2)
C(2)	59(3)	67(3)	43(3)	20(2)	10(2)	33(3)
C(1X)	47(3)	51(3)	44(4)	17(3)	9(3)	29(3)
C(2X)	54(4)	51(4)	53(4)	20(3)	10(3)	32(3)

4 Supplementary Reference

1. Kyte, J., Doolittle, R.F. *J. Mol. Biol.*,**157**, 105-132 (1982).
2. Bartoloni, M., Jin. X., Marcaida, M. J., Banha, J., Dibonaventura, I., Bongoni, S., Bartho, K., Gräbner, O., Sefkow, M., Darbre, T., Reymond, J. *Chem. Sci.* **6**, 5473–5490 (2015).
3. Wareham, R. S., Kilburn, J. D., Turner, D. L., Rees, N. H. & Holmes, D. S. *Angew. Chem. Int. Ed. Engl.* **34**, 2660–2662 (1996).
4. Knappe, T. A., Linne, U., Zirah, S., Rebuffat, S., Xie, X., Marahiel, M. *J. Am. Chem. Soc.* **130**, 11446–11454 (2008).
5. Reisberg, S. H., Gao, Y., Walker, A., Helfrich, E. J., Clardy, J., Baran, P. S. *Science* **367**, 458–463 (2020).
6. Nicolaou, K. C., Boddy, C. N. C. & Siegel, J. S. *Angew. Chem. Int. Ed. Engl.* **40**, 701–704 (2001).
7. McGibbon, R. T., Beauchamp, K. A., Harrigan, M. P., Klein,C., Swails, J. M., Hernández, C. X., Schwantes, C. R., Wang, L.-P., Lane, T. J., Pande, V. S. *Biophys. J.* **109**, 1528-1532 (2015).
8. Vranken, W. F., Boucher, W., Stevens, T. J., Fogh, R. H., Pajon, A., Llinas, M., Ulrich, E. L., Markley, J. L., Ionides, J., Laue, E, D. *Proteins: Structure, Function and Genetics*, **59**(4), 687–696 (2005).
9. Yilmaz, E. M., Güntert, P. *J Biomol NMR*. **63**, 21-37 (2015).
10. Pettersen, E. F., Goddard, T. D., Huang, C. C., Couch, G. S., Greenblatt, D. M., Meng, E. C., Ferrin, T. E. *J Comput Chem*. **25**(13):1605-12 (2004).
11. Yao, G., Knittel, C. H., Kosol, S., Wenz, M. T., Keller, B. G., Gruß, H., Braun, A. C., Lutz, C., Hechler, T., Pahl, A. Suessmuth, R. D. *J. Am. Chem. Soc.***143**, 14322-14331 (2021).
12. [a] Abraham, M. J., van der Spoel, D., Lindahl, E., Hess, B. GROMACS development team, (2019); [b] Van Der Spoel, D., Lindahl, E., Hess, B., Groenhof, G., Mark, A. E., Berendsen, H. J. *J. Comput. Chem.* **26**, 1701-1718 (2005); [c] Abraham, M. J., Murtola, T., Schulz, R., Páll, S., Smith, J. C., Hess, B., Lindahl, E. *SoftwareX* **1**, 19-25 (2015).
13. Hanwell, M. D., Curtis, D. E., Lonie, D. C., Vandermeersch, T., Zurek, E., Hutchison, G. R. *J. Cheminform.* **4**, 17 (2012).
14. Sousa da Silva, A. W., Vranken, W. F. *BMC Res Notes* **5**, 367-367 (2012).
15. Maier, J. A., Martinez, C., Kasavajhala, K., Wickstrom, L., Hauser, K. E., Simmerling, C. *J. Chem. Theory Comput.* **11**, 3696-3713 (2015).
16. Van Gunsteren, W. F., Berendsen, H. J. *Mol. Simul.* **1**, 173-185 (1988).
17. Hess, B., Bekker, H., Berendsen, H. J., Fraaije, J. G. *J. Comput. Chem.* **18**, 1463-1472 (1997).
18. Bussi, G., Donadio, D., Parrinello, M. *J. Chem. Phys.* **126**, 014101 (2007).
19. Parrinello, M., Rahman, A. *J. Appl. Phys.* **52**, 7182-7190 (1981).
20. Páll, S., Hess, B. *Comput. Phys. Commun.* **184**, 2641-2650 (2013).
21. Darden, T., York, D., Pedersen, L. *J. Chem. Phys.* **98**, 10089-10092 (1993).
22. Harris, C. R., Millman, K. J., van der Walt, S. J., Gommers, R., Virtanen, P., Cournapeau, D., Wieser, E., Taylor, J., Berg, S., Smith, N. J. *Nature* **585**, 357-362 (2020).

23. Yao, G., Kosol, S., Wenz, M.T., Irran, E., Keller, B. G., Trapp, O., Süssmuth, R. D. DOI: 10.5281/zenodo.6974777 (2022).
24. Shrake, A., Rupley, J. A. *J. Mol. Biol.* **79**, 351-371 (1973).
25. Neese, F., Wennmohs, F., Becker, U., Riplinger, C. *J. Chem. Phys.* **152**, 224108 (2020).
26. Bannwarth, C., Ehlert, S., Grimme, S. *J. Chem. Theory Comput.* **15**, 1652-1671 (2019).
27. [a] Becke, A. D. *J. Chem. Phys.* **96**, 2155-2160 (1992); [b] Lee, C., Yang, W., Parr, R. G. *Phys. Rev. B* **37**, 785 (1988); [c] Becke, A. D. *J. Chem. Phys.* **98**, 1372-1377 (1993); [d] Caldeweyher, E., Bannwarth, C., Grimme, S. *J. Chem. Phys.* **147**, 034112 (2017); [e] Weigend, F., Ahlrichs, R. *Phys. Chem. Chem. Phys.* **7**, 3297-3305 (2005).
28. Barone, V., Cossi, M. *J. Phys. Chem. A* **102**, 1995-2001 (1998).
29. Neese, F., Wennmohs, F., Hansen, A., Becker, U. *Chem. Phys.* **356**, 98-109 (2009).
30. Abraham, M. J., van der Spoel, D., Lindahl, E., Hess, B. GROMACS development team (2021)**.**
31. Zagrovic, B., Van Gunsteren, W. F. *Proteins* **63**, 210-218 (2006).
32. Tropp, J. *J. Chem. Phys.* **72**, 6035-6043 (1980).
